# Transgenic Overexpression of Myocilin Leads to Variable Ocular Anterior Segment and Retinal Alterations Associated with Extracellular Matrix Abnormalities in Adult Zebrafish

**DOI:** 10.3390/ijms23179989

**Published:** 2022-09-01

**Authors:** Raquel Atienzar-Aroca, Jesús-José Ferre-Fernández, Angel Tevar, Juan-Manuel Bonet-Fernández, María-José Cabañero, María-José Ruiz-Pastor, Nicolás Cuenca, José-Daniel Aroca-Aguilar, Julio Escribano

**Affiliations:** 1 Área de Genética, Facultad de Medicina de Albacete, Universidad de Castilla-La Mancha, 02006 Albacete, Spain; 2 Instituto de Investigación en Discapacidades Neurológicas (IDINE), Universidad de Castilla-La Mancha, 02006 Albacete, Spain; 3 Cooperative Research Network on Age-Related Ocular Pathology, Visual and Life Quality (OFTARED), Instituto de Salud Carlos III, 28029 Madrid, Spain; 4 Department of Physiology, Genetics and Microbiology, University of Alicante, 03080 Alicante, Spain

**Keywords:** myocilin, myoc, zebrafish, transgenic myoc, anterior segment alterations, retinal dysplasia, matricellular protein

## Abstract

Myocilin is an enigmatic glaucoma-associated glycoprotein whose biological role remains incompletely understood. To gain novel insight into its normal function, we used transposon-mediated transgenesis to generate the first zebrafish line stably overexpressing myocilin [Tg(actb1:myoc-2A-mCherry)]. qPCR showed an approximately four-fold increased myocilin expression in transgenic zebrafish embryos (144 hpf). Adult (13 months old) transgenic animals displayed variable and age-dependent ocular anterior segment alterations. Almost 60% of two-year-old male, but not female, transgenic zebrafish developed enlarged eyes with severe asymmetrical and variable abnormalities in the anterior segment, characterized by corneal limbus hypertrophy, and thickening of the cornea, iris, annular ligament and lens capsule. The most severe phenotype presented small or absent ocular anterior chamber and pupils, due to iris overgrowth along with dysplastic retinal growth and optic nerve hypertrophy. Immunohistochemistry revealed increased presence of myocilin in most altered ocular tissues of adult transgenic animals, as well as signs of retinal gliosis and expanded ganglion cells and nerve fibers. The preliminary results indicate that these cells contributed to retinal dysplasia. Visual impairment was demonstrated in all old male transgenic zebrafish. Transcriptomic analysis of the abnormal transgenic eyes identified disrupted expression of genes involved in lens, muscular and extracellular matrix activities, among other processes. In summary, the developed transgenic zebrafish provides a new tool to investigate this puzzling protein and provides evidence for the role of zebrafish myocilin in ocular anterior segment and retinal biology, through the influence of extracellular matrix organization and cellular proliferation.

## 1. Introduction

The *MYOC* gene encodes myocilin, a secreted glycoprotein build-up of 504 amino acids. The normal function of this protein is not well known, although its role in autosomal-dominant juvenile glaucoma, an optic neuropathy caused by apoptosis of retinal ganglion cells [1], is well established [2]. Myocilin, initially called Trabecular Meshwork Inducible Glucocorticoid Response (TIGR), was identified as an overexpressed protein in glucocorticoid-treated human trabecular meshwork cells [3]. Transcripts encoding myocilin were independently discovered by subtractive cDNA cloning in the human ciliary body [4,5] and in photoreceptor cells [6]. Kubota et al. denominated this protein myocilin based on the amino acid sequence similarity of its N-terminal region with myosin [6]. *MYOC* is expressed in some parts of ocular anterior segment, e.g., iris and trabecular meshwork [5,7,8,9], and the protein is also present in the aqueous humor [10,11], where it may be secreted from the ciliary body and the trabecular meshwork in association with exosomes [12,13]. Non-ocular tissues, such as skeletal and cardiac muscles [5], leukocytes and lymphoid tissues [14], also express this gene, and the protein is present in blood plasma [14].

Structurally, myocilin is organized into three different regions: the N-terminal part, which in the secreted protein is composed of two coiled-coil domains [15,16], and a leucine-zipper motif [5] located in the second coiled-coil; the central region containing a calpain II proteolytic site [11,17]; and the olfactomedin homologue C-terminal region [5], which is characteristic of the olfactomedin protein family. Olfactomedins are glycoproteins with roles in the nervous system, early development and hematopoiesis [18]. The olfactomedin domain of myocilin presents a five-bladed β-propeller globular structure [19] and is affected by most glaucoma-related mutations [5]. A singular quaternary structure has been reported for myocilin, consisting of a Y-shaped dimer-of-dimers [20]. The interaction of myocilin through its olfactomedin domain with proteins, such as SPARC, hevin [21,22] and fibronectin [23], suggests that myocilin might function as a matricellular protein [24], and as such, it may participate in regulating cell–matrix interactions, rather than being structural part of the extracellular matrix (ECM). The anti-adhesive properties of myocilin on trabecular meshwork cells [25,26] and on human circulating leukocytes [14] also support this concept. Myocilin undergoes an intracellular proteolytic cleavage releasing two similar size fragments that may be required to tune its molecular interactions [21,27,28].

Growing experimental evidence indicates that myocilin plays a role in different cell signaling pathways including Wnt [29,30], through which it can regulate biological processes, such as zebrafish sexual differentiation [31] and the ligand-mediated endocytosis of the G-protein-coupled receptor GPR-143 [32,33], which is involved in the pigmentation pathway taking place in the retinal pigment epithelium (RPE).

Loss-of-function, overexpression and/or misexpression of myocilin in mouse models have been used to investigate its role in glaucoma pathogenesis and the biological pathways in which this protein is involved. A myocilin knockout mouse revealed that this protein is not required for normal intraocular pressure or normal ocular morphology and suggested that disease-causing mutations in humans likely act by gain of function [34]. Transgenic mice expressing pathogenic variants of human or mouse myocilin reproduced glaucoma phenotypes observed in patients (i.e., elevated intraocular pressure, retinal ganglion cell death and axonal degeneration) and provided evidence for the role in the disease of endoplasmic reticulum stress induced by accumulation of the mutant protein [35,36,37,38,39]. However, other transgenic mouse lines expressing either mutant [40,41] or wild-type myocilin [41,42] did not develop glaucoma. In addition, the study of a transgenic Drosophila line also supported a role for aggregation of myocilin in the endoplasmic reticulum and activation of the unfolded protein response in myocilin-associated glaucoma [43]. On the other hand, overexpression of wild-type mouse myocilin by a transgenic mouse resulted in a 36% increased average size of muscle fibers and revealed that myocilin interacts with syntrophin, a component of dystrophin-associated protein complex, indicating that it is involved in muscle hypertrophy pathways [37].

Herein, we report the first *myoc* transgenic zebrafish model as an additional tool to investigate the function of this protein. Characterization of this transgenic line provides new evidence for the role of zebrafish myocilin as a regulator of ECM and cell proliferation in the anterior segment of the eye and the retina.

## 2. Results

### 2.1. Generation of Tg(actb1:myoc-2A-mCherry) Bicistronic F0 Zebrafish

The *myoc*-2A-mCherry plasmid was obtained as described in the Materials and Methods Section (Supplementary Figure S1A) and co-injected with transposase mRNA into the yolk of one-cell zebrafish embryos (Supplementary Figure S1B). As expected, most F0 animals showed mosaic mCherry fluorescence (Supplementary Figure S1C) and the offspring (F1) were screened by fluorescence microscopy to identify founders transmitting the *myoc*-2A-mCherry bicistronic protein. To increase the probability of obtaining fishes carrying reliable single transgene insertions with predictable Mendelian inheritance of the functional transgene, we carried out two consecutive outcrosses of transgenic animals with wild-type AB zebrafish (F1–F3, Supplementary Figure S1B). F3 animals showed an approximately 50% fluorescence segregation, supporting the successful integration of the transgene in the founder’s germline and the existence of a single genomic insertion in the obtained transgenic line. qPCR using as a template mRNA from 144 hpf F4 transgenic larvae showed an approximately four-fold *myoc* increase compared with the wild type (Supplementary Figure S1D), supporting transgene overexpression.

### 2.2. Mapping Transgene Integration Sites

To identify the Tol2-mediated genomic insertion site of the transgene, we used nested PCR. Tailfin genomic DNA was digested with *Alu*I to generate transgene-derived DNA fragments ending in the *Alu*I sequences located at the Tol-2 flanking sequences (Supplementary Figure S2A). Then the restriction fragments were self-ligated and amplified by PCR using two nested primer pairs specific for 3′- and 5′-Tol2 sequences (Supplementary Figure S2A, arrowheads). The amplification yielded characteristic bands (A5-1 and A5-2 5′ from the Tol2-5′-end and A3-1 and A5-3 from the Tol2-3′-end) that were different from those present in the wild-type genomic DNA used as a control (Supplementary Figure S2B). The bands amplified from each end correspond to self-ligation of different fragments that resulted from cleavage at different *Alu*I targets located in the proximity of the transgenic insertion sites. Sanger sequencing of the amplicons using the nested Tol-2 specific primers revealed genomic sequences surrounding the insertion site of the transgene, which, although they were not clean in all cases, identified a region from chromosome 11 in the proximity of the insertion site. Additional sequencing primers were designed using these sequences from chromosome 11 (Supplementary Figure S2A, arrows), and the new electropherograms revealed the 5′- and 3′-Tol2 sequences placed at the end of the transgenic construction, followed by an eight bp duplication (Supplementary Figure S2C, underlined sequence), which is characteristic of Tol2-mediated transposition [44]. The electropherograms also showed the genomic nucleotides flanking Tol2 sequences in the integration site. Blast analysis of the identified genomic sequences demonstrated that the integration site was located on chromosome 11, in an intergenic region upstream of the *tbl1xr1a* gene (Supplementary Figure S2D).

### 2.3. Phenotypic Characterisation of the Tg(actb1:myoc-2A-mCherry) Zebrafish Line

**Figure 1:**
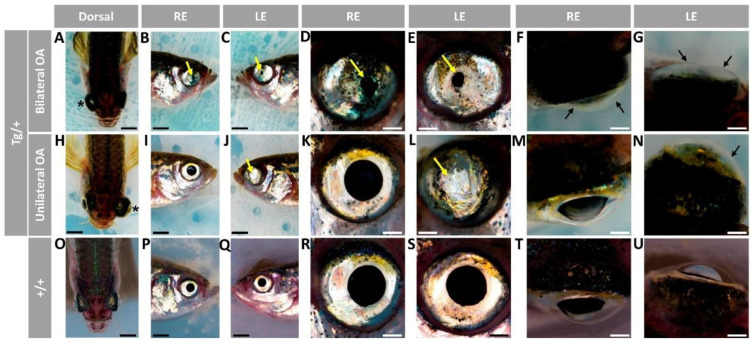
Variable ocular phenotypes in old (two-year-old) transgenic *myoc* zebrafish. Brightfield images of representative male *myoc* transgenic zebrafish with (**A**–**G**) bilateral or (**H**–**N**) unilateral ocular alterations. (**O**–**U**) A wild-type zebrafish of the same age is shown as a control. Yellow arrows: iris overgrowth and reduced or absent pupil; black arrows: cloudy corneas and reduced anterior chamber size; asterisk: enlarged eyeballs; scale bar in panels (**A**–**C**, **H**–**J**, and **O**–**Q**): 200 μm; scale bar in panels. (**D**–**G**, **K**–**N**, and **R**–**U**): 50 μm. LE: left eye. OA: ocular alterations; RE: right eye. Tg/+: transgenic. +/+: wild type.

We did not observe significant gross external macroscopic alterations in larvae (96 hpf) or adult (5, 7 and 11 months) *Tg(actb1:myoc-2A-mCherry)* F3 heterozygous zebrafish (data not shown). In addition, analysis of histological eye sections stained either with hematoxylin-eosin or Sirius red did not reveal any meaningful difference between transgenic and wild-type animals (data not shown). A total of 21 animals (14 males and 7 females) survived to the age of two years, and of them, 8 males (57.15%), but none of the females (Supplementary Figure S3) developed variable ocular phenotypes affecting the anterior segment and/or the eyeball. Three of the transgenic males presented bilateral iris overgrowth that resulted in small or absent pupils (Figure 1B–E,J,L, yellow arrows) with cloudy and, in some eyes, flattened corneas (Figure 1F–G, black arrows), suggesting the existence of corneal stroma abnormalities. Reduced anterior chamber size (Figure 1A,F,G) and unilaterally enlarged eyeballs (Figure 1A, asterisk) were also observed. The remaining five transgenic zebrafish showed similar unilateral iris, cornea and anterior segment alterations (Figure 1H–N), and three of them also exhibited unilaterally enlarged eyes (Figure 1H, asterisk). These alterations were not observed in control wild-type zebrafish of the same age and sex (Figure 1O–U).

**Figure 2:**
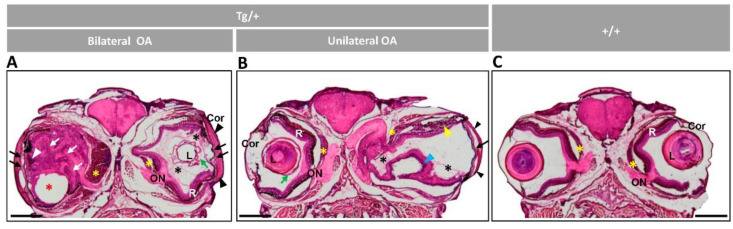
Histology of head sections of adult male transgenic zebrafish (two years old). Hematoxylin-eosin-stained tissue sections of transgenic zebrafish with (**A**) bilateral or (**B**) unilateral macroscopic ocular alterations. (**C**) Control tissue sections from wild-type zebrafish of the same age and sex. Black arrows: contacts between iris and cornea; black arrowheads: increased thickness of the corneal stroma; black asterisks: increased vitreous-like material; green arrows: folded and thickened lens capsule; blue arrowhead: overgrowth and folding of the retina; red asterisk: displaced lens: white arrows: hypertrophy of the retinal fiber layer and optic nerve; yellow arrowheads: abnormal photoreceptors; yellow asterisks: overgrowth of the choroid body. Scale bar: 800 µm. Cor: cornea; OA: ocular alterations; ON: optic nerve; L: lens; R: retina; +/+: wild type; Tg/+: transgenic.

Head sections of transgenic zebrafish with bilateral or unilateral ocular alterations were stained with hematoxylin-eosin for histological evaluation. A general observation of these preparations revealed remarkable corneal thickening and fusion between the cornea and iris (Figure 2A,B, black arrowhead and black arrow, respectively) and confirmed the presence of reduced or absent anterior chamber in the most severe ocular phenotypes (Figure 2A,B, black arrows), correlating with in vivo observations (Figure 1F,G,N). The lens capsule showed variable thickening and folding (Figure 2A,B, green arrows). The transgenic lens capsule presented more than two-fold increase in average thickness compared to the wild-type lens capsule (Supplementary Figure S4A). Sirius red staining confirmed these findings and demonstrated the intense red decoration in the corneal stroma and annular ligament (Supplementary Figure S5, arrowheads) and lens capsule (Supplementary Figure S5, green arrows), indicating increased collagen deposition. Large accumulations of vitreous-like material were present in some eyes (Figure 2A,B, black asterisks). Retinal pigment epithelial (RPE) cells at the iridocorneal angle were disorganized, with irregularly folded layers and invading the anterior part of the retinal mass (Figure 2A, white arrowhead). In addition, we observed variable neuroretinal alterations that in the most severe phenotype were characterized by a retinal mass occupying the vitreous cavity and displacing ventrally the lens (Figure 2A, red asterisk), associated with an extremely enlarged choroid body (Figure 2A, yellow asterisk). This unexpected retinal structure was apparently dominated by folded and hypertrophic extensions of the retinal nerve fiber layer (Figure 2A, white arrows), which converged in a hypertrophic optic nerve. Abnormal photoreceptors, retinal folding (Figure 2B, yellow and blue arrowheads, respectively) and the hypertrophic optic nerve (Figure 2A,B, ON) were present as well. Control wild-type zebrafish of the same age and sex did not show these features (Figure 2C).

**Figure 3:**
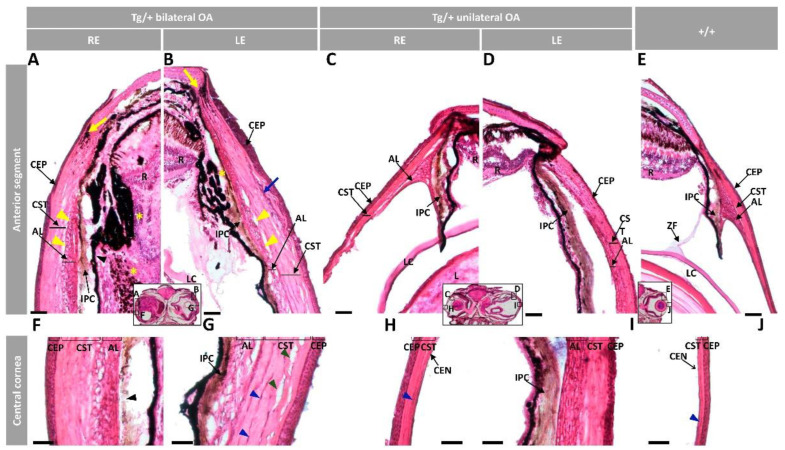
Histology of ocular anterior segment from old (two-year-old) *myoc* transgenic zebrafish. Hematoxylin-eosin-stained tissue sections of transgenic zebrafish with (**A**,**B**,**F**,**G**) bilateral or (**C**,**D**,**H**,**I**) unilateral macroscopic ocular alterations. (**E**,**J**) Control tissue sections from wild-type zebrafish of the same age and sex. Head tissue sections in the inserts indicate the localization of the images shown in the different panels. Scale bars: 50 µm. The images are representative of two individuals of each genotype. AL: annular ligament; CEN: corneal endothelium; CEP: corneal epithelium; CST: corneal stroma; R: retina; IPC: iris pigment cells; L: lens; LC: lens capsule; LE: left eye; OA: ocular alterations; RE: right eye; ZF: zonular fiber; black arrowhead: close contact between the hypertrophic annular ligament and iris; blue arrow: collagen cords; blue arrowheads: cavities in the CST; green arrowhead: keratocytes; yellow arrows: limbal melanocytes; yellow arrowheads: cavities between IPC and AL; yellow asterisk: increased RPE cells; Tg/+: transgenic; +/+: wild type.

Detailed microscopic examination of the ocular anterior segment at a higher magnification confirmed the abnormalities consisting of variable thickening of the corneal epithelium, corneal stroma and annular ligament, especially in the central cornea (Figure 3A–D and Figure S4B–D). Formation of large collagen cords in the corneal epithelium (Figure 3B, blue arrow) and presence of cavities between the corneal stroma and annular ligament (Figure 3A,B, yellow arrowheads) were also observed. In the central cornea we also observed areas of close contact between the hypertrophic annular ligament and iris (Figure 3F,G,I). In some eyes the corneal stroma also presented an apparently increased number of keratocytes and cavities (Figure 3G, blue and green arrowheads, respectively). The corneal limbus was also remarkably enlarged (Supplementary Figure S4E) and presented a likely expanded number of melanocytes (Figure 3A,B, yellow arrows). In the extreme phenotype, RPE cells accumulated in the iridocorneal angle, infiltrating the anterior retinal mass (Figure 3A,B, yellow asterisks). Wild-type-like eyes of transgenic zebrafish with unilateral ocular alterations did not show these features, although the corneal stroma was thickened compared with wild-type eyes (Figure 3C,H), which might correspond to an initial stage of ocular alterations. The described anomalies were not observed in the ocular anterior segment wild-type zebrafish of the same age and sex (Figure 3E,J).

**Figure 4:**
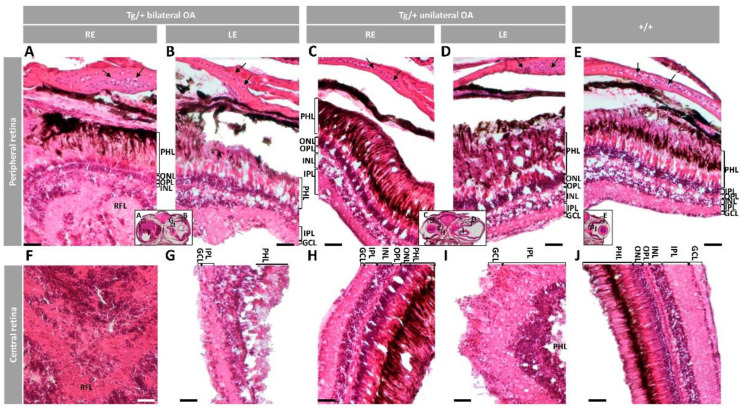
Histology of retinas from old (two-year-old) male *myoc* transgenic zebrafish. Tissue sections were stained with hematoxylin-eosin. Variable retinal disorganization and dysplasia, ranging from (**A**,**F**) the presence of a neuroretinal mass to (**B**,**D**,**G**,**I**) different degrees of photoreceptor degeneration and increased number of nuclei in different layers or (**C**,**H**) no evident alterations. Control wild type retina (**E**,**J**). Head tissue sections in the inserts indicate the localization of the images shown in the different panels. The images are representative of two individuals per phenotype. Arrows: chondrocytes in the scleral cartilage. Scale bars: 50 μm. Only clearly identifiable retinal layers are indicated. GCL: ganglion cell layer; IPL: inner plexiform layer; INL: inner nuclear layer; LE: left eye; OA: ocular alterations; OPL: outer plexiform layer; ONL; outer nuclear layer; PHL: photoreceptor layer; RE: right eye; RFL: retinal fiber layer; Tg/+: transgenic; +/+: wild type.

Histological examination of the retinas revealed variable alterations that in the most severe phenotype was characterized by a disorganized neuroretinal mass, invading the vitreous cavity, although some layers were recognizable in the peripheral retina (Figure 4A, RPE to inner nuclear layer, INL). Apparently, an increased nuclei number was present both in the inner plexiform layer (IPL) and the ganglion cell layer (GCL), along with retinal fiber layer (RFL) hypertrophy (Figure 4A). These preliminary observations, which require further confirmation, suggest the existence of retinal ganglion cell proliferation. Variable photoreceptor disruption as well as disorganized plexiform and nuclear layers were also observed (Figure 4B–D). Similarly, the central retina was highly degenerated, with disorganized photoreceptors, unstructured plexiform and nuclear layers and absence of the RPE (Figure 4F,G,I). However, the retinal layers were recognizable in some eyes (Figure 4H). The eyeball presented thickened scleral cartilage with increased number of chondrocytes and surrounding ECM in the most severe phenotypes (Figure 4A–D, arrows). These alterations were not observed in wild-type retinas (Figure 4E,J). Altogether these data show the existence of variable alterations in the ocular anterior segment and retina of old transgenic male zebrafish with characteristic hypertrophy and/or dysplasia and increased ECM deposition.

### 2.4. Immunohistochemical Analysis of Ocular Tissues

**Figure 5:**
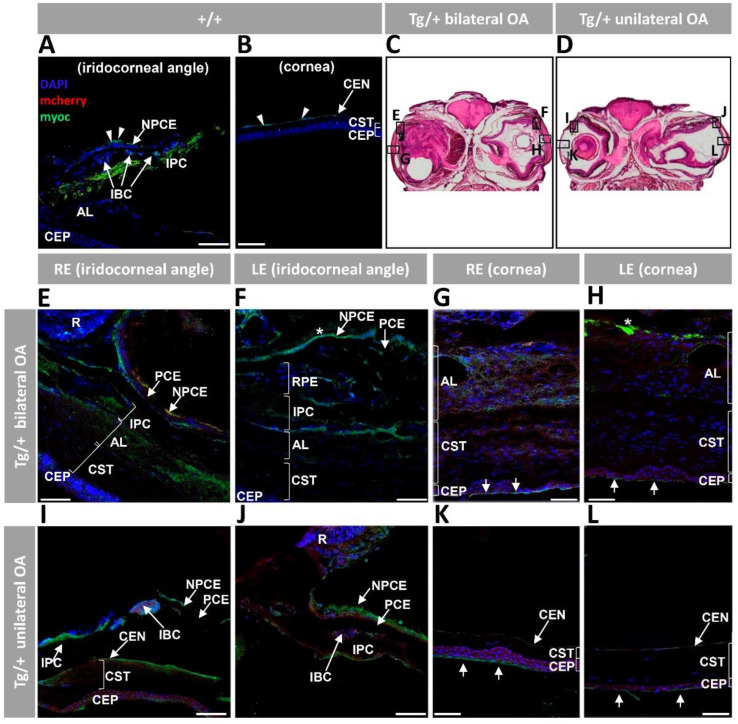
Fluorescent immunohistochemistry expression analysis of myocilin and mCherry in the anterior segment of old (two years) *myoc* transgenic zebrafish. A chicken anti-myocilin primary antibody was used to detected myocilin (green signal). Control wild type iridocorneal angle (**A**) and cornea (**B**). Representative head sectionsindicate the regions analyzed by immunohistochemistry in transgenic zebrafish with bilateral (**C**) or unilateral (**D**) ocular alterations. Anterior segment tissue sections from zebrafish with bilateral (**E**–**H**) or unilateral (**I**–**L**) ocular alterations. Asterisk in (**F**,**H**): myocilin immunoreactivity in the vitreous and iris stroma, respectively. Arrows in (**G**,**H**,**K**,**L**): myocilin immunoreactivity in the most superficial layer of the corneal epithelium. Scale bars: 50 μm; AL: annular ligament; CEN: corneal endothelium; CEP: corneal epithelium; CST: corneal stroma; IBC: iris blood cells; IPC; iris pigment cells; LE: left eye; NPCE: non-pigmented ciliary epithelium; OA: ocular alterations; PCE: pigmented ciliary epithelium; R: retina; RE: right eye; RPE: retinal pigment epithelial cells; +/+: wild type; Tg/+: transgenic.

To evaluate the correlation of ocular alterations with the presence of transgenic myocilin, we analyzed the bicistronic transgene expression in eye tissues of old transgenic zebrafish. Myocilin was detected by fluorescence immunocytochemistry using a chicken anti-myocilin antibody targeted against the N-terminal region of the human protein (TNT antibody) [7,31]. Fluorescence microscopy was employed to identify the presence of the reporter protein mCherry. In accordance with previous reports [31], analysis of the anterior segment of control wild-type eyes showed myocilin immunoreactivity in the non-pigmented ciliary epithelium (NPCE), iris pigment cells (IPC) (Figure 5A) and corneal endothelium (Figure 5B). Blood cells in the iris were also positive for myocilin immunolabelling (Figure 5A). Representative areas of the anterior segment were selected for the immunohistochemical analysis of transgenic zebrafish (Figure 5C,D). The eyes of transgenic animals presented clear myocilin signals in the altered tissues of the iridocorneal angle, i.e., NPCE, IPC, annular ligament and corneal stroma (Figure 5E,F,I,J). Blood cells in the iris also showed anti-myocilin staining (Figure 5I). Remarkably, the area where RPE cells accumulated (see Figure 3A,B, yellow asterisk) was positive for myocilin staining (Figure 5F, RPE). The central cornea of altered eyes showed variable and diffuse extracellular anti-myocilin labelling in the corneal stroma and annular ligament (Figure 5G,H) with areas of intense accumulation in the iris stroma (Figure 5H, asterisk). The anti-myocilin antibody also decorated the corneal endothelium and especially the most superficial layer of the corneal epithelium (Figure 5G,H,K,L, arrows). The vitreous was positive for myocilin immunoreactivity (Figure 5F, asterisk). Although mCherry fluorescence was not very intense, yellowish areas were detected in the NPCE (Figure 5E), showing co-localization of this protein with myocilin in this epithelial layer. In addition, a diffuse red background was present both in the altered annular ligament and corneal stroma (Figure 5G,H), as well as in the corneal epithelium (Figure 5G–I,K,L). The lack of precise anti-myocilin and mCherry signal overlapping in some places may be due to the different cellular fate of the two proteins (myocilin is a secreted protein and mCherry remains in the intracellular space). Chondrocytes and perichondrium of the scleral cartilage in wild-type zebrafish showed anti-myocilin decoration (Supplementary Figure S6A, arrowheads and arrows, respectively). Dorsal areas of transgenic eyeballs were selected for immunocytochemical analysis (Supplementary Figure S6C,D). The transgenic hypertrophic scleral cartilage presented increased anti-myocilin staining, along with weak mCherry fluorescence (Supplementary Figure S6E–H), showing that transgenic myocilin overexpression is associated with hypertrophy of this cartilage. Notably, DAPI staining revealed an increased number of nuclei in the stroma of both cornea (Figure 5G,H,K and Figure S4F) and annular ligament (Figure 5G,H and Figure S4G), IPC and the region of RPE accumulation (Figure 5F and Figure S4H), suggesting the existence of cellular proliferation in these structures. The specificity of fluorescent mCherry and anti-myocilin signals was supported by absence of mCherry labelling in wild-type tissues of the anterior segment (Figure 5A,B and Figure S6A), as well as lack of positive signals in the negative controls (Supplementary Figures S6B and S7A,B).

**Figure 6:**
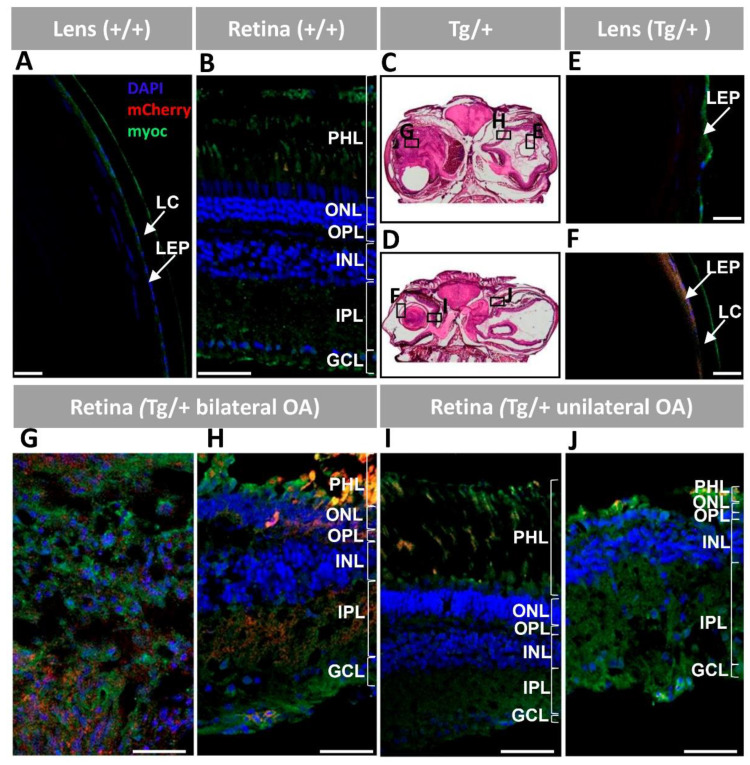
Fluorescent immunohistochemistry expression analysis of myocilin and mCherry in the lens and retina of old (two-year-old) *myoc* transgenic zebrafish. A chicken anti-myocilin primary antibody was used to detected myocilin (green signal). Control wild type lens (**A**) and retina (**B**). Representative head tissue sections in panels (**C**,**D**) indicate the regions analyzed by immunohistochemistry in transgenic zebrafish with bilateral (**C**) or unilateral (**D**) ocular alterations. Lens (**E**,**F**) and retina (**G**–**J**) tissue sections. Scale bars: 50 μm. GCL: ganglion cell layer; IPL: inner plexiform layer; INL: inner nuclear layer; LC: lens capsule; LEP: lens epithelium; OA: ocular alterations; OPL: outer plexiform layer; ONL; outer nuclear layer; PHL: photoreceptor layer; +/+: wild type; Tg/+: transgenic.

We also analyzed the correlation of bicistronic transgene expression with eye lens and retinal alterations. In control wild-type zebrafish, the lens epithelium and the external surface of the lens capsule (Figure 6A), as well as the retinal GCL, IPL and photoreceptors (Figure 6B), were labelled with the anti-myocilin antibody. These signals likely correspond to the endogenous protein. Representative areas of the lens and retina were selected for the immunohistochemical analysis of transgenic zebrafish (Figure 6C,D). The severely affected transgenic eyes presented clear anti-myocilin signals, together with mCherry fluorescence, in the lens epithelium (Figure 6E,F), confirming the expression of the transgenic protein. The anti-myocilin antibody also painted the superficial layer of the lens capsule (Figure 6F). On the other hand, the retinal mass exhibited an intense and diffuse anti-myocilin labelling in the hypertrophic retinal fiber layer (Figure 6G). Retinal regions where the different layers were recognizable presented GCL and IPL diffuse myocilin immunolabelling (Figure 6H–J). Photoreceptors also showed anti-myocilin immunoreactivity, particularly in the area likely corresponding to the external segment of rods (Figure 6H–J). Diffuse mCherry signals coinciding with myocilin immunolabeling were present in the retinal mass (Figure 6G), photoreceptors and IPL (Figure 6H), supporting the expression of the transgenic proteins. The wild-type retina lacked mCherry signals, although weak photoreceptor autofluorescence was seen (Figure 6B), and the negative controls did not show any signal (Supplementary Figure S7C,D), indicating that the observed mCherry and anti-myocilin fluorescent labelling in the lens and retina were specific. These data clearly show the correlation between the presence of transgenic myocilin and lens and retinal alterations.

**Figure 7:**
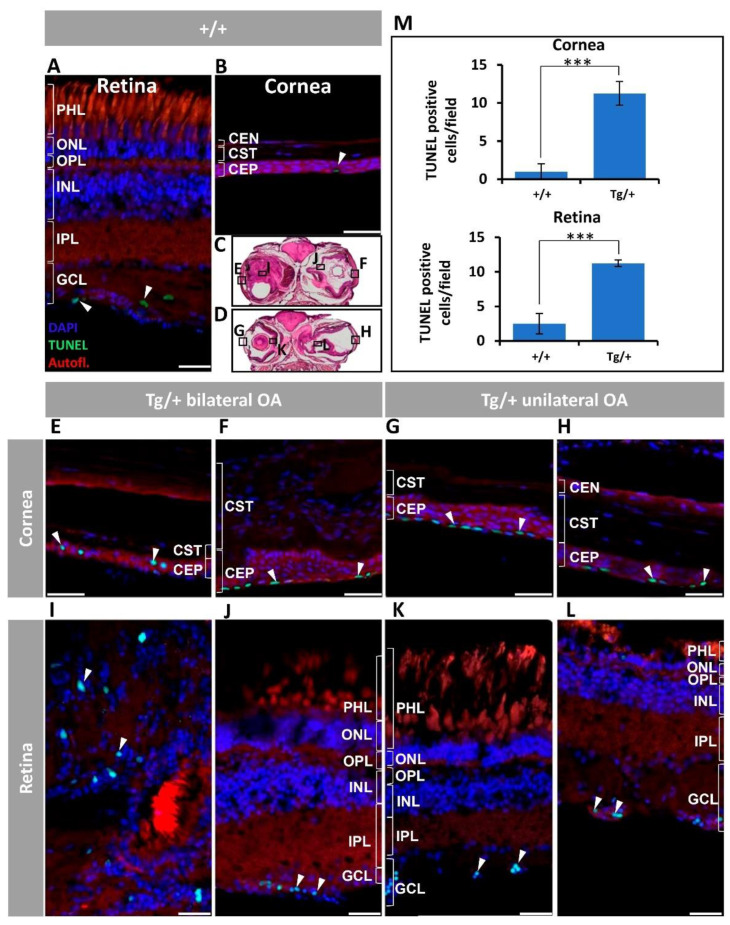
Corneal epithelium and retinal ganglion cell apoptosis in old (two-year-old) *myoc* transgenic zebrafish. Apoptosis was assessed using terminal dUTP nick-end labelling (TUNEL) of fragmented DNA. (**A**) Wild-type retina and (**B**) cornea. (**C**,**D**) Representative transgenic head tissue sections indicate the regions analyzed by immunohistochemistry. (**E**–**H**) transgenic cornea. (**I**–**L**) Transgenic retina. Scale bar in (**A**,**I**–**L**): 25 μm. Scale bar in (**B**,**E**–**H**): 50 μm. (**M**) Quantification of TUNEL positive cells. Four microscopic fields per eye were analyzed (n = four eyes). ***: *p* < 0.001, Student’s *t*-test. White arrowheads: TUNEL-positive cells. Autofl.: tissue autofluorescence used for image contrast and anatomical reference. CEP: corneal epithelium; CST: corneal stroma; CEN: corneal endothelium; GCL: ganglion cell layer; IPL: inner plexiform layer; INL: inner nuclear layer; OA: ocular alterations; OPL: outer plexiform layer; ONL; outer nuclear layer; PHL: photoreceptor layer; Tg/+: transgenic; +/+: wild type. The images are representative of the results observed in two fishes of each genotype.

To evaluate the presence of apoptosis associated with the ocular alterations of transgenic zebrafish, we carried out a TUNEL assay. Both control wild-type retina and cornea presented a reduced number of TUNEL positive cells in the GCL and the most superficial layer of the corneal epithelium (Figure 7A,B, arrowheads). However, representative areas of transgenic zebrafish (Figure 7C,D) presented an increased number of positive cells in the corneal epithelium, mainly located in the most superficial layer (Figure 7E–H, arrowheads), but also in the interior epithelial layers of the most severely affected eyes (Figure 7E, arrowheads). Apoptotic cells were not observed in any other part of the anterior segment. TUNEL-positive cells were also detected in the retinal mass (Figure 7I, arrowheads), as well as in the GCL of transgenic eyes with less severe phenotypes (Figure 7J–L). The quantitative analysis revealed a significant 5- to 10-fold increase in apoptotic cells in the corneal epithelium and retina of transgenic zebrafish, compared with the corresponding tissues of wild-type animals (Figure 7M). The specificity of the TUNEL assay was supported by positive nuclear staining in the cornea and retina of the positive controls (Supplementary Figure S8A,C) and the absence of signals in the corresponding negative controls (Supplementary Figure S8B–D).

**Figure 8:**
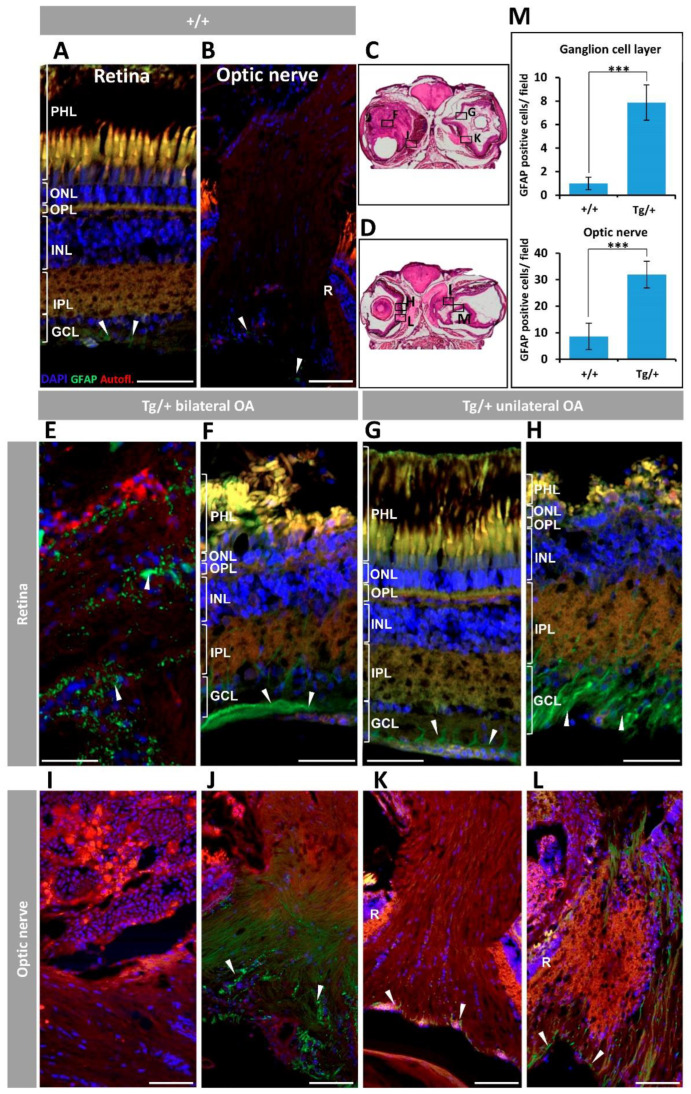
Retinal and optic nerve gliosis in old (two years) *myocilin* transgenic zebrafish. A rabbit anti-GFAP primary antibody was used to detected Müller glial cells. (**A**) Wild-type retina and (**B**) optic nerve. (**C**,**D**) Representative transgenic head tissue sections indicating the regions analysed by immunohistochemistry. (**E**–**H**) Transgenic retina. (**I**–**L**) Transgenic optic nerve. (**M**) Quantification of GFAP-positive cells in the ganglion cell layer and optic nerve. Four microscopic fields per eye were analyzed (n = four eyes). ***: *p* < 0.001, Student’s *t*-test. Arrowheads: GFAP labelling of Müller cells. Scale bars: 50 μm. Autofl.: tissue autofluorescence used for image contrast and anatomical reference; GCL: ganglion cell layer; IPL: inner plexiform layer; INL: inner nuclear layer; OA: ocular alterations; OPL: outer plexiform layer; ONL; outer nuclear layer; PHL: photoreceptor layer; R: retina. Tg/+: transgenic; +/+: wild type. The images are representative of the results observed in two zebrafish of each type.

Retinal and optic nerve gliosis was also assessed immunohistochemically using an anti-GFAP antibody. As we anticipated, control wild-type retina and optic nerve showed weak GFAP immunoreactivity, mainly localized in the GCL and also in the optic nerve surface (Figure 8A,B, arrowheads). In contrast, the retinas from transgenic animals exhibited increased retinal GAFP immunoreactivity dominated by a granular pattern in the retinal mass that did not associate with any identifiable cell layer (Figure 8E, arrowheads). In eyes with better-preserved retinal structure strong anti-GFAP signals were mainly localized in the GCL (Figure 8F–H, arrowheads), supporting Müller cell activation. Variable GFAP immunoreactivity was observed in the optic nerve of transgenic animals, ranging from undetectable or weak (Figure 8I,K, respectively) to intense (Figure 8J,L). Although it has been found that GFAP is expressed only in Müller glial cells of the zebrafish retina [45], we did not observe the typical morphology of these cells in the altered transgenic zebrafish, which might be due to severe structural and cellular retinal alterations present in these animals. Anti-GFAP staining was negative in the most affected optic nerve (Figure 8I), which might indicate a complete loss of Müller cells because of advanced optic nerve degeneration. The specificity of the GFAP immunoreactivity was supported by the absence of signals in the negative control (Supplementary Figure S9).

**Figure 9:**
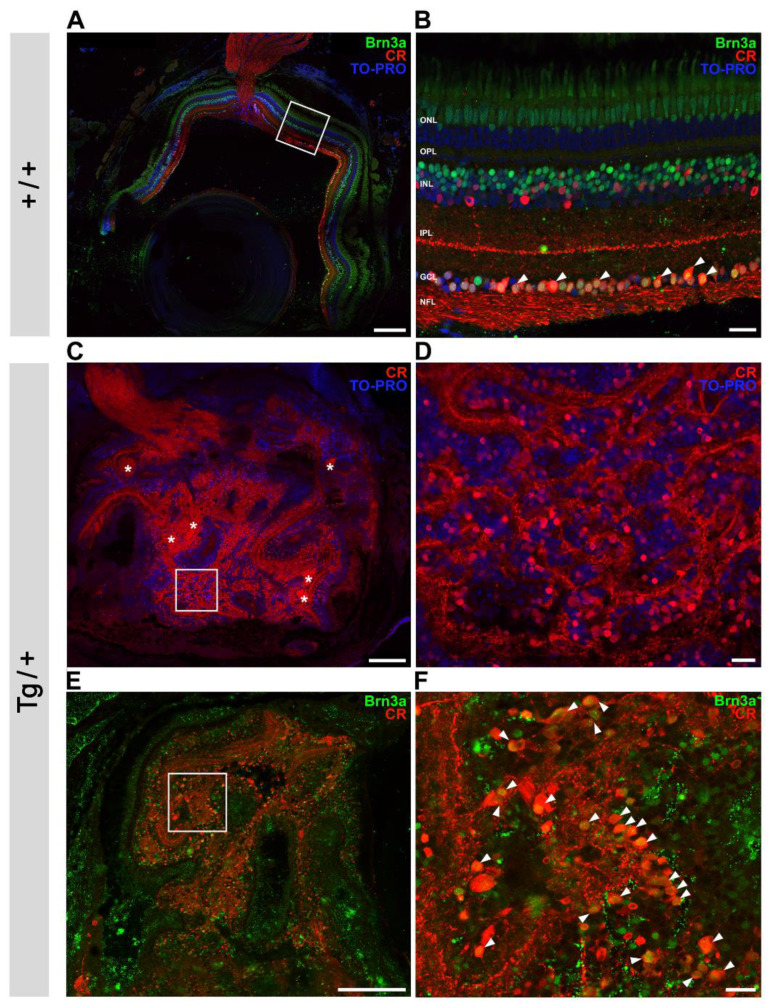
Increased number of retinal ganglion cells in the retinal mass present in the most severe phenotype of old (two-year-old) *myocilin* transgenic zebrafish. Overview and detailed images of (**A**,**B**) wild-type zebrafish eye and (**C**–**F**) *myocilin* transgenic zebrafish eye. Images in (**B**,**D**,**F**) are details indicated by white boxes in (**A**,**C**,**E**), respectively. (**A**,**B**) Double immunostaining for calretinin (CR), a calcium-binding protein, and the transcription factor Brn3a was used for the identification of ganglion cells (arrowheads). Optic nerve fibers were immuno-positive for calretinin. TO-PRO-3-iodide was used for nuclei staining. (**C**–**F**) Asterisks: axon bundles. Scale bars in (**A**,**C**,**E**): 200 µm; scale bars in (**B**,**D**,**F**): 20 µm. ONL: outer nuclear layer; OPL: outer plexiform layer; INL: inner nuclear layer; IPL: inner plexiform layer; GCL: ganglion cell layer; NFL: nerve fiber layer; Tg/+: transgenic; +/+: wild type.

To identify the contribution of retinal ganglion cells to the retinal mass that characterized the most severe transgenic zebrafish ocular phenotype, double immunolabeling against calretinin [46] and Brn3a [47] was used. Control retinas showed clear calretinin immunoreactivity in the retinal ganglion cell layer and optic nerve fibers. Additionally, a subpopulation of amacrine and bipolar cells in the inner nuclear layer showed positive immunoreactivity for calretinin (Figure 9A,B). Most ganglion cells, in addition to bipolar cells and cone photoreceptors, showed Brn3a immunoreactivity (Figure 9A,B). Ganglion cells were identified by their double immunolabeling for calretinin and Brn3a (Figure 9B, arrowheads). TO-PRO-3-iodide was used for nuclei staining. In accordance with the previous histological analysis (see Figure 2A), overview of the immunostained transgenic eyes showed the loss of retinal organization (Figure 9C,E). In addition, several axon bundles (asterisks) and increased calretinin-positive cells were observed (Figure 9C,D). The abundance of double-positive cells indicated the expansion of retinal ganglion cells in the dysplastic retina (Figure 9E,F, arrowheads). These data support the contribution of increased retinal ganglion cells and nerve fibers to the highly unstructured retina of zebrafish overexpressing myocilin. No positive signals were observed in the negative controls, showing the specificity of the immunolabeling (Supplementary Figure S10).

### 2.5. Visual Function

The visual function of the transgenic zebrafish line was evaluated using the social preference test described in materials and methods. We tested a total of 13 available transgenic *myoc* male zebrafish of which six presented wild-type-like ocular phenotypes and seven showed uni- or bilateral ocular alterations. Four male wild-type zebrafish of the same age and sex were used as controls. Interestingly, all transgenic animals showed a significant reduction of the time spent at the window proximal to the social stimulus (approximately four times shorter for wild-type-like transgenic animals and 10 times shorter for transgenic zebrafish with ocular alterations; Supplementary Figure S11). These results indicate that all old transgenic zebrafish has impaired visual function, even those with wild-type-like ocular phenotypes.

### 2.6. Ocular Transcriptomic Profile

To characterize ocular gene expression changes associated with *myoc* overexpression and ocular alterations in old transgenic zebrafish, we carried out RNAseq as described in the Materials and Methods Section. The purified mRNA from each experimental group was pooled to minimize the effect of individual variability. From a total of 39987 coding RNAs and multiple non-coding polyadenylated RNAs identified in the transcriptomic analysis, we excluded 18,954 genes with zero counts, selecting 21,033 genes for differential expression analysis. Pearson’s coefficient used to assess the similarity between samples indicated a high similarity among samples, with the highest value obtained between the two wild-type replicas (Supplementary Figure S12A). On the other hand, comparison of DEG patterns (fold change ≥ 2 and raw *p*-value < 0.05) by hierarchical clustering analysis also showed the higher similarity between the two wild-type replicas (Supplementary Figure S12B), indicating that many detected wild-type gene expression patterns were reproducible.

To identify DEGs in the altered eyes of transgenic zebrafish we compared gene expression of each transgenic ocular transcriptome with each of the two independent biological replicas of the ocular wild-type transcriptome (Tg/+ OA vs. +/+1 and Tg/+ OA vs. +/+2). We found that an average of 2422 genes were significantly up-regulated (fold change > 2 and raw *p* < 0.05) and 2652 genes were significantly down-regulated (fold change < −2 and raw *p* < 0.05, Supplementary Figure S13). As expected, we found a significant increased myocilin expression in the transgenic eyes (2.83-fold; *p* = 1.3 × 10^−5^).

**Figure 10:**
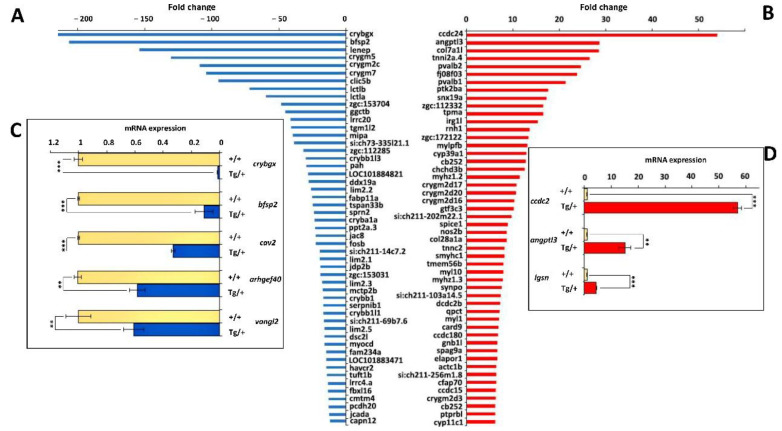
Top-50 DEGs in altered eyes of old (two-year-old) male *myoc* transgenic zebrafish. (**A**) Down- and (**B**) up-regulated genes identified by high-throughput RNA sequencing with significant differences coinciding in the comparison with the two wild-type ocular transcriptomes (Tg/+ OA vs. +/+1 and Tg/+ OA vs. +/+2). Confirmation by qRT-PCR of differential gene expression of selected (**C**) down- and (**D**) up-regulated genes. Aliquots of RNA preparations used for transcriptomic analyses were used as templates in qRT-PCR. Values represent the average of six experimental replicas. **: *p* < 0.01; ***: *p* < 0.001, Student’s *t*-test.

We selected for further analyses the significant top-50 down- and up-regulated genes that coincided in the two comparisons of the ocular transgenic transcriptome with each of the two wild-type ocular transcriptomes (Figure 10 and Supplementary Tables S2 and S3). The absolute gene expression differences were remarkable for eyes with ocular alterations, ranging from approximately 10-fold to more than 200-fold for down-regulated genes (Figure 10A and Supplementary Table S2) and from 6-fold to more than 50-fold for up-regulated genes (Figure 10B and Supplementary Table S3).

To evaluate the reliability of the identified DEGs, we evaluated by qRT-PCR expression differences of some selected representative genes. First, we selected the two most up- (*coiled-coil domain containing 24*, *ccdc24;* and *angiopoietin-like protein 3*, *agptl3*) or down-regulated (*crystallin beta gamma X*, *crybgx;* and *beaded filament structural protein 2*, *phakinin*, *bfsp2*) genes. Interestingly, three of these genes encoded lens proteins. The qRT-PCR results confirmed the differential expression differences, although absolute fold-change values were smaller than those obtained in the transcriptomic analysis (Figure 10C,D and Supplementary Table S4). This discrepancies between RNAseq and qRT-PCR are not unusual and may be explained by the methodological differences of the two procedures.

Second, we also re-evaluated by qRT-PCR a group of DEGs that were not in the top-50 DEGs but presented absolute expression difference values higher than 2 and were considered functionally interesting. These genes included *lgsn* (*lengsin*, *lens protein with glutamine synthetase domain*), *cav2* (*caveolin 2*), *arhgef40* (*rho guanine nucleotide exchange factor 40*) and *vangl2* (*VANGL planar cell polarity protein 2*). *Lgsn* is expressed in the lens [48], *cav2* is a glaucoma-related gene [49] like *myoc*, *vangl2* encodes a wnt-related protein [50] and *arhgef40* plays a role in cell adhesion [51]. The qRT-PCR also confirmed the RNAseq results (Figure 10C,D and Supplementary Table S4).

Next, to unveil functional relationships in the group of top-100 DEGs (top-50 up- plus top-50 down-regulated genes), we carried out a comprehensive enrichment analysis using Epistemic AI, an artificial intelligence web-based software platform [52], and three different databases (Elsevier pathway collection, BioPlanet 2019 and KEGG pathway database). The results revealed that at least 12 of these genes (12% of top DEGs) encoded lens- and cataract-related proteins, e.g., beta-, gamma- and beta-gamma-crystallins; lens intrinsic protein; lens epithelial protein; and lactase-like proteins (Supplementary Table S5). At least six muscle-related genes (6% of top DEGs, including myosin, actin, tropomyosin and troponin), were also significantly overexpressed in the abnormal eyes of old male transgenic zebrafish overexpressing myocilin (Supplementary Table S5). In addition, genes involved in metabolism of endogenous sterols (*cyp39a1* and *cyp11c1*) and folate (*zgc:153031* and *zgc:153031*), visual cycle (*zgc:112332* and *zgc:112332*) and leukocyte transendothelial migration (*myl10*, *mylpfb*, and *ptk2ba*) were also enriched among these DEGs.

A complementary functional enrichment evaluation of the same group of DEGs using the gene ontology enrichment analysis web tool ShinyGO [48] showed similar results. The different categories that resulted from this analysis were classified into two major functional groups: (1) ocular- and lens-related development and (2) skeletal muscle contraction (Supplementary Table S6). These groups were composed, respectively, of the lens- and muscle-related genes identified in the previous analysis. Raw RNAseq data used to identify DEGs are shown in Supplementary Tables S7 and S8.

Interestingly, two alpha collagen genes, *col7a1l* and *col28a1a*, were up-regulated in altered eyes of transgenic zebrafish. *Col7a1l* was the third most overexpressed gene (Figure 10B and Supplementary Table S3). These changes support the increased deposition of extracellular matrix observed in the altered eyes of transgenic zebrafish.

### 2.7. Confirmation of the Ocular Phenotypes in a New Generation of Transgenic Myoc Zebrafish

**Figure 11:**
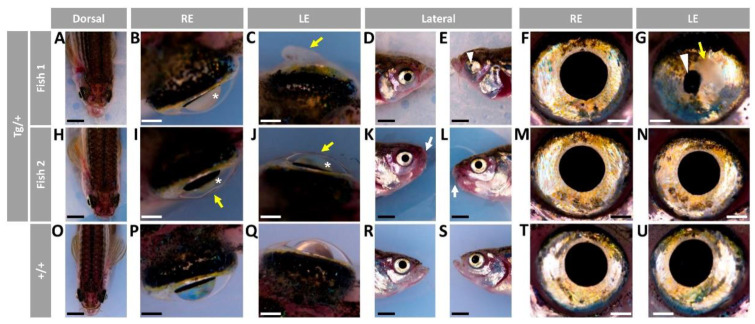
Variable ocular phenotypes in adult (13-month-old) F4 transgenic *myoc* zebrafish. Brightfield images of (**A**–**N**) representative *myoc* F4 transgenic male zebrafish with ocular alterations and (**O**–**U**) wild-type as a control. Asterisks: cloudy corneas; yellow arrows: corneal hyperplasia; white arrowhead: reduced pupil size. Scale bars in (**A**,**D**,**E**,**H**,**K**,**L**,**O**,**R**,**S**): 200 μm; Scale bars in (**B**,**C**,**F**,**G**,**I**,**J**,**M**,**N**,**P**,**Q**,**T**,**U**): 50 μm; LE: left eye; RE: right eye; Tg/+: transgenic; +/+: wild type.

We obtained a new generation (F4) of heterozygous transgenic animals to corroborate the ocular alterations identified in the old transgenic zebrafish and to determine more precisely their onset. Due to the advanced age of F3 transgenic zebrafish, the F4 generation was obtained by in vitro fertilization. To that end, we employed the sperm of three wild-type-like transgenic F3 males and oocytes from two wild-type female zebrafish. The male and female gametes were pooled. In total, 18 out of 40 embryos were positive for mCherry fluorescence, indicating that they were transgenic. These animals were examined for macroscopic and histological (hematoxylin-eosin and Sirius red staining) ocular alterations at the ages of 5 (four individuals), 7 (six individuals) and 13 months (seven individuals). Macroscopic ocular abnormalities were not evident until the 13th month of life. The seven zebrafish that remained alive at this age were males, and in six of them (85.7%) the lens was variably cloudy, indicating the existence of cataract (Figure 11A–C,I,J, asterisk). The lens abnormalities were not visible in lateral views (Figure 11D–G,K–N). Three siblings showed additional variable alterations of the anterior segment of the eye, affecting the cornea and iris. Corneal phenotypes ranged from apparently normal (Figure 11B) to corneas with large hyperplasia (Figure 11C, yellow arrow), thickened (Figure 11I, yellow arrows) and flattened areas (Figure 11J, yellow arrows). Iris overgrowth with reduced and irregular pupil (Figure 11E,G, white arrowhead), and decreased anterior chamber size (Figure 11E,G, white arrowhead) was also present in the eye with corneal hyperplasia (Figure 11G, black arrowhead). In addition, this same zebrafish also presented shortened and outwardly curved operculums (Figure 11D,E). Two transgenic individuals (28%) presented lower jaw shortening (Figure 11H,K,L, white arrow). All these alterations were not present in control wild-type zebrafish of the same sex and age (Figure 11O–U).

**Figure 12:**
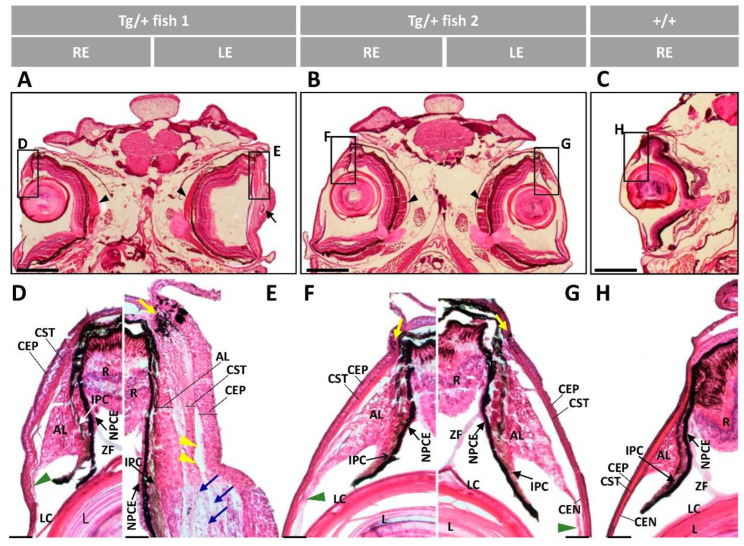
Histology of the anterior ocular segment of F4 adult male transgenic zebrafish (13 months). Tissue sections were stained with hematoxylin-eosin. Scale bars in (**A**–**C**): 800 µm. Scale bars in (**D**–**H**): 50 µm. The images are representative of five transgenic zebrafish. AL: annular ligament; CEP: corneal epithelium; CST: corneal stroma; CEN: corneal endothelium; R: retina; IPC: iris pigment cells; L: lens; LC: lens capsule; NPCE: nonpigmented ciliary; ZF: zonular fiber; LE: left eye; RE: right eye; Tg/+: transgenic; +/+: wild type; black arrow: deposit of amorphous material; black arrowheads: enlarged choroid body; blue arrows: increased keratocytes; green arrowhead: altered annular ligament: yellow arrows: limbal melanocytes; yellow arrowheads: cavities between the AL and CST.

Histological analysis of hematoxylin-eosin-stained eye tissue sections from five transgenic and two 13-month-old wild-type zebrafish (control) confirmed the main variable alterations of the anterior ocular segment detected in their F3 siblings. A general observation revealed variable alterations in the anterior segment of three animals, with no evident retinal alterations (Figure 12A,B). The lens capsule and epithelium were apparently unaffected at this stage, and no other significant lens abnormalities were observed in hematoxylin-eosin-stained sections from zebrafish with lens clouding (Figure 12A,B). Detailed histological examination of the anterior segment clearly showed variable degrees of thickened corneal epithelium, corneal stroma and annular ligament in all eyes (Figure 12D–G). The most severe phenotype presented an extremely hypertrophic anterior segment, with remarkable increased number of cells and cell layers of the corneal epithelium, strong corneal stroma and annular ligament thickening, iris overgrowth with absence of the anterior chamber and deposit of amorphous material in the corneal stroma (Figure 12A, LE and Figure 12E). Additional features of this severe phenotypes were the presence of abundant cells, probably keratocytes, in the thickened corneal stroma (Figure 12E, blue arrows), formation of cavities between the corneal epithelium and stroma, as well as in the contact points between the cornea and iris (Figure 12E) (Figure 12B,G), alongside enlargement of corneal limbus, increased number of limbal melanocytes (Figure 12E–G, yellow arrows) and thickening and hypertrophy of the NPCE (Figure 12E). Bilateral enlargement of the choroid body was also detected in these animals (Figure 12A,B, black arrowheads). Wild-type zebrafish did not show any of these ocular alterations (Figure 12C,H) indicating that they are specific of transgenic animals.Overall, these results reveal that 13-month-old F4 transgenic *myoc* zebrafish anticipate most of the main variable macroscopic and histologic ocular alterations identified in old (two-year-old) F3 transgenic zebrafish. In addition, the ocular phenotypes of these transgenic zebrafish show that the alterations begin in the anterior segment and in more advance stages they extend to the retina.

**Figure 13:**
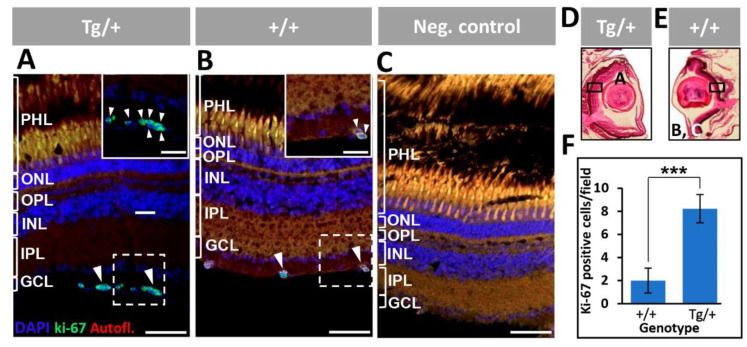
Proliferation of retinal ganglion cells in adult (13-month-old) *myocilin* transgenic zebrafish. (**A**,**B**) An anti-Ki-67 primary antibody was used to detect proliferating cells. Digital magnification of the positive cells indicted with white dotted boxes are shown in the inserts. (**C**) The negative control consisted of tissue sections incubated only with the secondary antibody. (**D**,**E**) Black rectangles in two representative eye sections indicate the location of microscopic fields shown in the different panels. (**F**) Quantification of Ki-67-positive cells. Four microscopic fields per eye were analyzed (n = four eyes). ***: *p* < 0.001, Student’s *t*-test. Scale bars: 50 μm. Arrowheads: Ki-67-positive cells. Scale bars: 50 μm. GCL: ganglion cell layer; IPL: inner plexiform layer; INL: inner nuclear layer; OPL: outer plexiform layer; ONL: outer nuclear layer; PHL: photoreceptor layer. The images are representative of the results observed in two fishes of each genotype.

Immunodetection of the cell proliferation marker Ki-67 [53] was used to determine the existence of dividing cells in the eyes of both adult (13-month-old) and old (two-year-old) transgenic zebrafish. Irregularly distributed groups of Ki-67-positive cells were identified in the retinal ganglion cell layer of 13-month-old transgenic zebrafish (Figure 13A). In contrast, wild-type zebrafish of the same age presented a small number of isolated Ki-67 immunoreactive cells in this retinal layer (Figure 13B), and the signals were not seen in the negative control (Figure 13C), indicating that they were specific. Representative areas of the retina were selected for the immunohistochemical analysis of transgenic and wild-type zebrafish (Figure 13D,E, respectively). The quantitative analysis showed an approximately four-fold increased number of Ki-67-positive cells in the retinal ganglion cell layer of transgenic animals, compared with wild-type zebrafish (Figure 13F). These results indicate that retinal ganglion cell proliferation begins at least at the age of 13 months and provide further support for the idea that these cells contribute to the severe retinal alterations detected in old zebrafish overexpressing myocilin.

No other cell proliferation differences were observed in ocular tissues of adult transgenic zebrafish. Ki-67 immunohistochemistry of old transgenic zebrafish eyes (twoyears) did not reveal significant differences with the wild-type eyes (Supplementary Figure S14), showing that at this age there was not a detectable increased cellular proliferation in the hypertrophic anterior segment and retina.

## 3. Discussion

The biological function of the glaucoma-associated protein myocilin remains poorly known. Both in vitro and in vivo (knockout and transgenic) models developed over more than 20 years have partially elucidated its biological function. Zebrafish can be used as a model organism to study the function of human myocilin because of the relatively good evolutionary conservation of these two orthologue genes. In fact, the *myoc* gene has four exons, whereas its human orthologue consists of three exons. Both genes present 37.2% nucleotide identity in their coding regions and encode proteins with a relatively well-conserved olfactomedin domain that presents 45% amino acid sequence identity, although the N-terminal-coiled coils of the human protein are not predicted in zebrafish myocilin [31]. On the other hand, overexpression and/or misexpression of wild-type gene products represent a powerful tool to identify biological pathways in which the corresponding genes are involved, and that may remain undetected by loss-of-function analysis [54]. To obtain new clues on myocilin’s biological role, in this study we generated, to the best of our knowledge, the first reported transgenic zebrafish line overexpressing myocilin. The established zebrafish line integrated the bicistonic transgene [*Tg(actb1:myoc-2A-mCherry)*] on chromosome 11, in an intergenic region upstream of the *tbl1xr1a* gene, resulting in an approximately four-fold myocilin overexpression compared with wild-type zebrafish.

Taking into account the expression of *myoc* in human and zebrafish ocular tissues [5,7,31] and the role of this gene in glaucoma; in this initial study we focused our analyses on ocular phenotypes associated with myocilin overexpression. Larvae and young adult transgenic zebrafish did not manifest any detectable macroscopic or histological ocular alterations. This finding was not surprising since previous reports have shown that vertebrate animal models under standard conditions do not develop ocular alterations as a result of either *myoc* knockout loss-of-function [31,34] or transgenic myocilin overexpression [42]. Nevertheless, elevated expression of this gene in the invertebrate Drosophila led to ocular alterations [43,55]. Interestingly, at the age of 13 months, we detected the presence of variable and incompletely penetrant ocular phenotypes in the anterior segment of transgenic zebrafish, affecting predominantly the cornea, annular ligament iris and lens. The most severe anterior segment abnormalities were characterized by enlargement of the corneal limbus, remarkable thickening of both corneal epithelium and stroma and overgrowth of the annular ligament and iris, which in the most severe phenotype resulted in reduced size of the anterior chamber and pupil. The ECM of most of these tissues was hypertrophic and, in some cases, showed striking thickening of the corneal stroma with the presence of cysts containing amorphous collagen deposits associated with abundant keratocytes. Expanded stromal keratocytes might indicate a defect in the limbal stem cell niche, the place of corneal and stromal cells’ formation in mature animals [56]. At this age, we also found hypertrophic NPCE and signs of retinal alterations consisting of variable nerve fiber layer thickening and increased ganglion cell proliferation. These changes might precede the severe dysplastic retinal alterations present in old transgenic zebrafish. Although different degrees of lens clouding were observed in alive transgenic zebrafish at this age, no significant histological alterations were detected with hematoxylin-eosin staining. An additional feature was the presence of variable choroid body hypertrophy. These ocular anomalies were also present in two-year-old male transgenic zebrafish, which in some eyes also showed increased collagen deposition and hypertrophy of the lens capsule. Moreover, the scleral cartilage exhibited increased number of chondrocytes and ECM, and very abundant vitreous material was associated with in the most severe phenotypes. The reduced number of 13-month-old transgenic zebrafish precluded a systematic follow-up of the phenotype progression until the age of two years. Further work is required to assess the evolution of ocular alterations between 13 and 48 months. Overall, these results show that myocilin overexpression associates with severe ECM alterations in different regions of the eye and increased number of ocular cells involved in ECM synthesis, i.e., keratocytes, epithelial lens cells, scleral cartilage chondrocytes and NPCE cells, in accordance with its role as a matricellular protein.

The variable retinal alterations associated with in vivo myocilin overexpression were characterized by areas of retinal degeneration. The most severe phenotype presented a remarkable retinal overgrowth that resulted in disorganization of the retinal layers and invasion of the vitreous cavity and optic nerve hypertrophy. Preliminary immunohistochemistry of calreticulin and Brn3a supports that ganglion cells may play key roles in these dysplastic retinal alterations. Analysis of the cell proliferation marker Ki-67 did not identify dividing cells in the dysplastic retina, indicating the slow progression of the phenotype and/or that the phenotype approached its final stage. However, a significant increased number of proliferating cells (Ki-67 positive) was observed in the retinal ganglion cell layer of 13-month-old transgenic zebrafish, suggesting that over-proliferation of these cells begins at least at this time, and supporting the contribution of the GCL to the phenotype. These findings need further confirmation.

The observed phenotypic variability might reflect, at least partially, different developmental stages of ocular alterations, which starting in the anterior segment of the eye may extend to the retina and the posterior pole. We hypothesize that the evolution of the phenotype may differ between the two eyes in the same zebrafish, depending on distinct exposure to environmental factors, generating intraindividual variability. In line with this hypothesis, individual genetic and/or environmental factors could generate inter-individual variability in ocular alterations.

The up-regulation of the intermediate filament protein GFAP, which is a cellular marker for retinal injury [57], and the expansion of Müller cells demonstrated the existence of severe retinal damage and gliosis in the transgenic zebrafish line overexpressing myocilin. Increased apoptosis in the GCL associated with retinal alterations further supported the role of this retinal layer in the phenotype. Functional evaluations demonstrated that these retinal alterations resulted in important visual impairment of transgenic zebrafish. Remarkably, transgenic zebrafish with no apparent ocular alterations also manifested visual loss, indicating the existence of molecular changes that affect sight before the damage is detectable at the histological and macroscopic levels. Future investigations are needed to determine molecular mechanisms leading to the initial loss of vision associated with myocilin overexpression.

Curiously, many of the described anterior segment defects resemble those caused by loss-of-function of the long form of the zebrafish gene *crumbs2b* (*crb2b-lf*), which are characterized by variable and incompletely penetrant expansion of the iris and tissues of the iridocorneal angle, resulting in small pupils, increased number of corneal stromal keratocytes, altered corneal endothelium and expanded lens capsule [58]. Crumbs are apical transmembrane proteins involved in epithelial organization and cell polarity processes [59], and its dysfunction is associated with loss of cell polarity and adhesion, increased early retinal apoptosis, disruption of lamination [60] and variable retinal degeneration [61,62]. The crumbs protein complex also coordinates multiple downstream signaling pathways, such as Notch and Hippo pathways, with roles in different developmental processes including cell self-renewal, proliferation, differentiation, mitosis and apoptosis [63,64]. Overall, these data may suggest a possible mechanism to explain the observed phenotypes, i.e., as a matricellular protein, myocilin overexpression might impair cell adhesion through the crumbs complex, contributing to the observed retinal alterations. In this line, it is also interesting that different reports have provided evidence on the cell adhesion role of myocilin, although probably by different mechanisms [14,65]. In line with this idea, it has been proposed that olfactomedin domains facilitate protein–protein interactions, intercellular interactions and cell adhesion [18]). Although all these data suggest a functional link between myocilin and crumbs pathways, further investigations are required to assess this hypothesis.

Another interesting finding was that adult male, but not female, transgenic zebrafish showed ocular alterations, indicating that long-term interplay of male physiological factors with overexpressed myocilin is required for developing the ocular alterations present in transgenic zebrafish. In this line, our previous work has proposed a role for myocilin in zebrafish sex determination [31]. Nevertheless, because of the relatively small sample of transgenic zebrafish, we cannot completely rule out a sampling sex bias in these observations. Thereby, additional work is required to confirm the result.

Our transcriptomic analysis revealed that many top DEGs in the altered eyes of transgenic zebrafish were characteristic of the lens, muscle and ECM. Genes involved in metabolism, inflammation, photoreceptor physiology and cell division were also differentially expressed, showing that these processes were affected by in vivo myocilin overexpression. Nevertheless, it could be difficult to determine whether these changes are cause or effect of the ocular phenotypes.

Crystallins differentially expressed in the transgenic eyes are predicted to be structural constituents of the eye lens, with roles in lens development in camera-type eyes and visual perception (https://zfin.org/, accessed on 10 January 2022), and are expressed in the lens epithelium [66]. Human orthologs of some of DEGs identified in this study, including *mipa* (major intrinsic protein of lens fiber, also known as aquaporin 0 or *aqp0*) and *bfsp2* (beaded filament structural protein 2, phakinin) are implicated in cataracts [67,68], and therefore, its dysregulated expression might contribute to lens cloudiness present in the eyes of some adult transgenic zebrafish. Mipa is a water channel and the most abundant protein in the cell membrane of lens fiber [69], and bfsp2 participates in intermediate filament organization in the eye lens [70]. Disruption of lens cell differentiation and interactions promoted by overexpression of two other lens genes, *lgsn* (*lengsin*) and *lenep* (*lens epithelial protein*), with roles in these biological processes [49,71], may influence the lens phenotypes. Interestingly, alpha crystallins, which are small heat shock proteins [72] that protect other proteins against stress-induced aggregation [73,74], were not found into the top DEGs. In addition, the only two alpha crystallins, *cryaa* and *cryabb*, that were differentially expressed were down-regulated with expression fold changes of −3.8 and −3.6, respectively. These data indicate that the detected lens phenotypes are not connected with cellular stress induced by overexpression of the transgenic protein.

The muscular-related DEGs encoded proteins that were either structural (*myl1* and *myl10*) or regulatory (myosin light chain, phosphorylatable, fast skeletal muscle b, *mylpfb*) constituents of the myosin light chain. Regulatory proteins of muscle contraction, such as troponin C2 (*tnnc2*), troponin I type 2a (skeletal, fast), tandem duplicate 4, (*tnni2a.4*) and alpha-tropomyosin (*tpma*), were also components of this group. Interestingly, two of these genes (*myl10* and *mylpfb*), along with protein tyrosine kinase 2 beta, a (*ptk2ba*), also belonged to functional enriched group related with leukocyte transendothelial migration (Supplementary Table S5), in line with the reported role of myocilin in adhesion of human leukocytes to endothelial monolayers [14]. These data indicate the possible existence of muscular phenotypes associated with myocilin overexpression in zebrafish. In accordance with this concept, it has been reported that the average size of muscle fibers of transgenic mice overexpressing myocilin increased by 36% relative to controls, suggesting that intracellular myocilin plays a role as a regulator of muscle hypertrophy pathways, acting through the components of dystrophin-associated protein complex [37]. Although in this study we did not evaluate non-ocular alterations, further investigations are required to assess the presence of muscular abnormalities associated with myocilin overexpression in zebrafish.

Several DEGs identified putative functional connections of myocilin with metabolism of folate and aldosterone, as well as sterols. Two folate-related genes were underexpressed in the eyes of transgenic zebrafish: phenylalanine hydroxylase (*pah*) and zgc:153031. The latter gene was identified by epistemic as orthologous to human dihydrofolate reductase and dihydrofolate reductase 2 (*DHFR* and *DHFR2*). On the other hand, *Cyp11c1* and *cyp39a1*, which are involved in sterol metabolism, were overexpressed. The former gene encodes a CYP450 enzyme that mainly catalyzes the formation of cortisol and the zebrafish androgen 11-Ketotestosterone [75,76]. Interestingly, *cyp11c1* is also up-regulated in a *myoc* knockout zebrafish line, which differentiates all individuals as males [31]. These data indicate the existence of a possible functional linkage between *cyp11c1* and *myoc* and the male-associated phenotypes observed in this study. The second gene, *cyp39a1*, is involved in bile acid biosynthesis [77] and cholesterol homeostasis [78]. These data reveal interesting connections of myocilin with biological processes, although additional experimental work is essential for a complete functional interpretation.

Overexpression of *spice1* (spindle and centriole associated protein 1), a gene required for centriole duplication and mitotic chromosome congression [79], supports the existence of increased cell proliferation and dysplasia in the altered eyes of the transgenic zebrafish.

Some of the gene expression changes detected in the altered eyes of transgenic zebrafish may be consequence or response to inflammation and tissular damage. In fact, the overexpressed genes *irg1l* (immunoresponsive gene 1-like) and *nos2b* (nitric oxide synthase 2b, inducible) are involved in inflammatory response to different insults [80,81,82]. Other interesting findings were difficult to interpret. For instance, two up-regulated genes, zgc:112332 and zgc:112332, were identified by epistemic as orthologous to human retinol dehydrogenases 11 and 12 (*RDH11* and *RDH12*) and human *LOC118142757*, respectively. The latter gene encodes a transcript resulting from readthrough between neighbor genes *GUCA1ANB* (*GUCA1A* neighbor) and a *GUCA1A* (guanylate cyclase activator 1A), which is translated into the same protein as GUCA1A (www.genecards.org, accessed on 11 January 2022). These data indicate that zgc:112332 and zgc:112332 play roles in the visual cycle, and their up-regulation in the transgenic zebrafish might be a consequence of photoreceptor alterations present in the eyes of transgenic zebrafish.

Given the evolutionary differences between the zebrafish and human proteins, further research is required to confirm whether the effects of myocilin overexpression in zebrafish may have a general biological meaning or if they are limited to this species. One limitation of the present study was the relatively small number of two-year-old transgenic zebrafish with ocular alterations. Having this in mind and to minimize the effect of individual gene expression variability, we pooled six altered eyes obtained from three old F3 siblings. Therefore, the RNA sample used for transcriptomics represents the average gene expression in three independent biological replicas. A parallel approach was followed with the two wild-type ocular RNA preparations used as reference. qPCR analysis of selected genes supported that gene expression differences detected in the transcriptomic analysis were reliable. On the other hand, two-year-old male wild-type zebrafish used as controls were bred in parallel with F3 transgenic animals, but they were not siblings of the F3 transgenic animals. Thus, additional replication of transcriptomic analysis, using wild-type siblings of transgenic zebrafish, would contribute to firmly demonstrate the identified gene expression differences.

## 4. Materials and Methods

### 4.1. Animals

Wild-type AB zebrafish (*Danio rerio*) were maintained at 28 °C with a 14 h on/10 h off light cycle and were fed a standard diet according to established protocols [83]. Zebrafish embryos were raised at 28 °C in E3 medium (5 mM NaCl; 0.17 mM KCl; 0.33 mM CaCl_2_; 0.33 mM MgSO_4_ and 0.0001% methylene blue, pH 7.2). Larvae and adult fishes were anesthetized with 0.02% and 0.04% tricaine methanesulfonate (#886-86-2, MS222, Sigma-Aldrich, St. Louis, MO, USA), respectively, and immobilized in 3% methylcellulose solution for analysis and photography.

### 4.2. Zebrafish DNA Extraction

Genomic DNA (gDNA) was isolated from whole zebrafish embryos (24 and 96 h post fertilization, hpf) using the HotSHOT method [84]. Tissue samples were incubated with 20 µL of base solution (25 mM KOH, 0.2 mM EDTA) at 95 °C for 30 min in a thermal cycler (BIORAD C100, BIORAD, Hercules, CA, USA), and then 20 µL of neutralization buffer (40 mM TrisHCl, pH 5) was added.

### 4.3. Plasmid Construction Entry Clones and Microinjection of Zebrafish Embryos

Entry vectors and the bicistronic construct to generate the transgenic zebrafish line expressing myocilin and mCherry under the constitutive promoter of beta actin were obtained using the MultiSite Gateway system (Invitrogen, Carlsbad, CA, USA) and the tol2kit plasmids [85], following the manufacturer’s indications. Briefly, a zebrafish *myoc* cDNA clone (Bioscience, ref: IRBOp991C0979D) was amplified using the following primers: 5′-GGGGACAAGTTTGTACAAAAAAGCAGGCT TCCCCAACATGTGGTTTTTAGC-3′ and 5′-GGGGACCACTTTGTACAAGAAAGCTGGGTCCTCCTGCTTGCCAAGTCTCA-3′. These two oligonucleotides contained the adapter primer sequences *att*B1 and *att*B2, respectively, which are underlined in the above nucleotide sequences. The PCR product was cloned by recombination into the *att*P-containing pDONR221 vector (Invitrogen), using BP Clonase (Invitrogen). The recombinant DNA was transformed into One Shot Mach1 competent cells (Invitrogen), and transformed cells were grown in the presence of kanamycin to obtain the middle entry plasmid (pME)-containing zebrafish *myoc* cDNA. Plasmid p5E-bactin2, containing 5.3 kb of the bactin2 promoter, as well as plasmids pME-myoc and p3E-P2A-mCherry, containing the viral peptide P2A and mCherry cDNA, were recombined and cloned into the destination vector pDestTol2pA using LR clonase (10134992, Invitrogen). Competent cells were transformed with the recombinant DNA and grown in the presence of ampicillin to obtain the plasmid containing the whole construct flanked by the Tol2 recombination sites.

One-cell stage zebrafish embryos were co-injected with 15 pg of the construct plasmid and 300 pg of the in vitro synthesized capped RNA (AM1340, mMESSAGE mMACHINE™ SP6, Ambion, Austin, TX, USA) of Tol2 transposase [86]. Fluorescent embryos expressing mCherry were selected and raised into adulthood, and they were screened to obtain the F0 founder fish carrying the bicistronic transgene in the germ line.

### 4.4. Nested PCR

Identification of the transgene integration site was carried out by nested PCR [87]. Pools of 30 F4 zebrafish embryos (144 hpf) positive for mCherry fluorescence were employed to purify genomic DNA using the Wizard SV Genomic DNA Purification System (A2360, Promega, Madison, WI, USA), following the manufacture’s recommendations. Overall, 800 ng of genomic DNA were digested for 16 h at 37 °C with *Alu*I (ER0011, ThermoFisher Scientific, Waltham, MA, USA) in Tango buffer. The samples were incubated for 10 min at 70 °C to inactivate restriction enzymes. Digested DNA (25 ng) was self-ligated with T4 DNA ligase (EL0011, ThermoFisher Scientific) overnight at 16 °C. Each half of the ligated sample (25 µL) was used to determine the 5′- or 3′ junction by two PCR rounds, using nested primers. The nested primers used to amplify the 5′ junction were: first round, Tol2-5′/f1 (5′-AGTACTTTTTACTCCTTACA-3′) and Tol2-5′/r1 (5′-GATTTTTAATTGTACTCAAG-3′); second round, Tol2-5′/f2 (5′-TACAGTCAAAAAGTACT-3′) and Tol2-5′/r2 (5′-AAGTAAAGTAAAAATCC-3′) [86]. Nested primers employed to amplify the 3′ junction were: first round, Tol2-3′/f1 (5′-TTTACTCAAGTAAGATTCTAG-3′) and Tol2-3′/r1 (5′-CTCCATTAAAATTGTACTTGA-3′); second round: Tol2-3′/f2 (5′-ACTTGTACTTTCACTTGAGTA-3′) and Tol2-3′/r2 (5′-GCAAGAAAGAAAACTAGAGA-3′) [86]. Two primers derived from chromosome 11 sequences, were used to confirm the transgenic insertion site: 5′-GATTAATTTTGGCGTTATGAG-3′ (forward) and 5′-GAATGTGAACAGGAAAAAGA-3′ (reverse). The PCR consisted of an initial step of 95 °C for 2 min followed by 30 amplification cycles (95 °C for 15 s; 48 °C for 30 s; 72 °C for 2 min). The nucleotide sequence of the PCR products was determined by automatic Sanger sequencing.

### 4.5. Quantitative Reverse Transcription PCR (qRT-PCR)

qRT-PCR was carried out as previously described [31]. RNA was isolated using the RNeasy Minikit (#74104, Qiagen, Germantown, MD, USA) and treated with RNase-free DNase I according to the manufacturer’s instructions, from pools of 50 zebrafish larvae (144 hpf, two independent biological replicas) or from a pool of altered eyes (six eyes) obtained from three adult male transgenic zebrafish (two years). As a control of ocular RNA, we used RNA from two independent biological replicas of an equivalent eye pool (six eyes) from three two-year-old wild-type male zebrafish. cDNA synthesis was caried out using the RevertAid First-Strand cDNA Synthesis Kits (#K1622, Thermo Fisher Scientific, Waltham, MA, USA) and the purified RNA as a template. mRNA expression relative to *ef1α* mRNA was determined using the 2^−∆∆Ct^ method [88] using the primer pairs described in Supplementary Table S1.

### 4.6. Zebrafish Tissue Samples

Adult transgenic and wild-type zebrafish heads were fixed overnight in 4% PFA and cryoprotected for two days at 4 °C in 30% sucrose/PBS 0.1 M (Dulbecco, X0515-500C). Thereafter, zebrafish heads were embedded in 10% porcine gelatin with 15% sucrose and stored at −80 °C. A cryostat (Leica CM3050 S, Leica Ltd., Wetzlar, Germany) was used to obtain serial cryosections (14 µm).

### 4.7. Fluorescence Immunohistochemistry

Fluorescence immunohistochemistry was carried out as previously described [31], using tissue sections from two zebrafish of each phenotype. Briefly, tissue sections (14 μm) were incubated with the following primary antibodies: chicken anti-myocilin (TNT, 1:150) [31], mouse anti-GFAP (1:100) (sc-33673, Santa Cruz Biotechnology, Dallas, TX, USA) and rabbit anti-ki67 (1:150) (GeneTex, Irvine, CA, USA)]. The corresponding secondary antibodies were, respectively, Cy2-conjugated donkey anti-chicken IgY (1:1000), Cy2-conjugated donkey anti-mouse (1:1000) and Cy2-conjugated donkey anti-rabbit IgY (1:1000) (Jackson ImmunoResearch, West Grove, PA, USA). For double calretinin and Brn3a immunolabeling, tissue sections were incubated overnight at room temperature with a mix of anti-Brn3a (1:500, Santa Cruz, sc-31984) and anti-calretinin (1:500, SWANT, CG1) in phosphate buffer (PB) with 0.1% of Tritón-X and 10% donkey serum. Sections were rinsed 3 times for 10 min in 0.1 M PB and incubated for 1 h with a proper secondary mix (anti-rabbit Alexa-555 and anti-goat Alexa-633, Molecular Probes, Eugene, OR, USA). Secondary antibody controls were made to test the specificity of the immunolabeling.

The In Situ Cell Death Detection Kit, Fluorescein (11684795910, Roche Diagnostics, Mannheim, Germany), was employed for TUNEL apoptotic cell death detection in tissue sections, following the manufacturer’s instructions. Positive controls were carried out as previously described [31]. Two to four animals from each experimental group were used for the microscopy analyses. Four tissue sections (14 μm) per fish were employed for each technique, and four random fields per tissue section were examined. The nuclei were stained either with DAPI (4′,6-diamidino-2-phenylindole, D8417, Sigma-Aldrich) at a 1:100 dilution in immunobuffer (10% fetal bovine serum, 1% dimethyl sulfoxide and 1% Triton X-100 in PB) for 2 min at room temperature as the last step of the process, or with TO-PRO-3 iodide (R37113, Invitrogen) at a 1:1000 dilution for 1 h at room temperature. These fluorophores were incubated together with the secondary antibody used in the different immunodetections. Tissue sections were mounted in Fluorescent Mounting Medium and visualized using an LSM710 Zeiss (Carl Zeiss, Jena, Germany) confocal microscope and the Zen (blue edition) software (Carl Zeiss) for image acquisition and analysis.

### 4.8. Histological Staining

Hematoxylin-eosin staining of histological sections of adult *myocilin* transgenic fish was carried out as previously described [31]. Head adult zebrafish sections were stained with Picro-Sirius Red (365548-5G, Sigma Aldrich) solution for 1 h, washed two times with 0.5% acetic acid for 5 min, dehydrated with 100% ethanol and washed with xylol at room temperature. The slides were mounted with Cytoseal (8311-4, Thermo Scientific, Waltham, MA, USA) Optical microscopy was carried out with a Nikon Eclipse-Ti (Nicon Corporation, Tokyo, Japan) microscope and the NIS-Elements software (Nikon) for image acquisition and analysis.

### 4.9. Visual Function Assay

The test employed to evaluate vision relies on the social preferences of zebrafish [89]. We used a large tank divided in two parts by a plate with a small transparent window. Fifteen wild-type zebrafish were introduced in one part of the tank, and the fish to be evaluated was situated in the other part. The experiments were conducted in the morning under constant illumination and water temperature (27 °C). All zebrafish swam and roamed freely all time. The fish to be tested was gently released into the tank and acclimatized for 30 min, then fish movements were recorded for 10 min with a camera and tracked using Tracker software (Open Source Physics, California, CA, USA). The time spent near the window proximal to the social stimulus was measured in three intervals of 1 min.

### 4.10. Ocular Transcriptome Analysis by RNAseq

RNA was extracted from a pool of six eyes with macroscopic alterations obtained from three transgenic siblings of the F3 generation (two-years old males), as described earlier [90]. Six eyes from three wild-type zebrafish of the same sex and age were treated in parallel as controls. We were able to carry out two independent biological replicas of wild-type eyes, but only one of transgenic eyes. The purified mRNA from each experimental group was pooled to minimize the effect of individual variability. Duplicates of RNA samples were outsourced to Macrogen Next-Generation Sequencing Division (Macrogen) for high-throughput sequencing. Libraries were generated using the TruSeq Stranded mRNA LT Sample Prep Kit (Illumina, Foster City, CA, USA), which captures both coding RNA and multiple forms of polyadenylated non-coding RNA. Paired-end sequencing (150 bp) was performed using a NovaSeq 6000 System (Illumina). Trimmomatic 0.38 [91] was used to remove bases with low-quality and adapter sequences. Reads were mapped to the zebrafish genome reference (GRCz11) with HISAT2 aligner [92]. The expression profile was calculated as read count and normalization value, which is based of transcript length and depth of coverage. DEG analysis of the myoc Tg vs. wild-type zebrafish eyes was performed using reads per kilobase of transcript per million mapped reads (RPKM). Genes with a fold change ≥ 2.0 and a *p*-value < 0.05 in the comparisons of the transgenic transcriptome with each of the two biological replicas of wild-type transcriptomes were considered as DEGs (Tg *myoc* OA vs. WT1 and Tg *myoc* OA vs. WT2). Functional gene enrichment analysis of DEGs was performed using the Epistemic Artificial Intelligence web-based software platform [52] and ShinyGO [93].

### 4.11. Statistics

Student’s *t*-test was employed for evaluation of statistical comparisons between groups using the SigmaPlot 12.0 software (Systat Software Inc., San Jose, CA, USA).

## 5. Conclusions

To the best of our knowledge, in this study, we report the first *myoc* transgenic zebrafish line. Characteristic anterior segment and retinal alterations appeared in adult male transgenic animals correlating with overexpression of the transgenic protein. These phenotypes were associated with disruption of ECM and altered expression of genes involved in lens, muscular and ECM activities, which may be cause or effect of the phenotype. This study provides further support for the function of myocilin in matricellular activities influencing cellular growth and tissular organization, at least in zebrafish. In addition, our results shed new insights into the complex biological activities of this fascinating and puzzling protein.

## Data Availability

Data are contained within the article or Supplementary Materials.

## Supplementary Materials

The following supporting information can be downloaded at: https://www.mdpi.com/article/10.3390/ijms23179989/s1.
