# Supplementary material for: Transgenic Overexpression of Myocilin Leads to Variable Ocular Anterior Segment and Retinal Alterations Associated with Extracellular Matrix Abnormalities in Adult Zebrafish
Source: Int J Mol Sci. 2022 Sep 1;23(17):9989. doi: 10.3390/ijms23179989 (PMC9456529; doi:10.3390/ijms23179989)
Supplement: Supplementary file 1 [file ijms-23-09989-s001.zip › ijms-1869389-supplementary.pdf]

Supplemental Materials

Transgenic overexpression of myocilin leads to variable ocular anterior segment and retinal alterations associated with extracellular matrix abnormalities in adult zebrafish

Raquel Atienzar-Aroca[1,2], Jesús-José Ferre-Fernández[1], Angel Tevar[1,2], Juan-Manuel Bonet-Fernández[1,2,4,], María-José Cabañero[1,2,4], María-José Ruiz-Pastor[3,4], Nicolás Cuenca[3,4], José-Daniel Aroca-Aguilar[1,24] and Julio Escribano[1,2,4]*

[1]Área de Genética, Facultad de Medicina de Albacete, Universidad de Castilla-La Mancha, Albacete 02006, Spain

[2]Instituto de Investigación en Discapacidades Neurológicas (IDINE), Universidad de Castilla-La Mancha, Albacete 02006, Spain

[3]Department of Physiology, Genetics and Microbiology, University of Alicante, Alicante 03080, Spain

[4]Cooperative Research Network on Age-Related Ocular Pathology, Visual and Life Quality (OFTARED), Instituto de Salud Carlos III, Madrid 28029, Spain

*Correspondence: julio.escribano@uclm.es

Supplementary Table S1. Primers used in RT-qPCR.

Gene	Forward primer (5'→3')	Reverse primer (5'→3')	Amplicon size (pb)
angptl3	TCACAAGAGGCCAGAAAACCAG	CTCCCTCCTCTGGATGACAGT	119
arhgef40	CCGTCTGAACTCTGCCTTT	GTTGGGTCGATTCGTCTGAT	106
bfsp2	GCCGAGACAGAATCGATCA	CCTCCAGCTTTCCGATAACA	118
cav2	GAGGTTCAGGACAGAGATCCTA	AGCATGACTACCAATCCAAACT	120
ccdc24	TGTTGCTGCTCACTCTGAGG	TGTTGCCCAGTGCATAACTT	105
crybgx	CGACTGGACCTGACCTCTGA	CCCCTGGAAGTTGTCATGTT	120
ef1α	CTGGAGGCCAGCTCAAACAT	ATCAAGAAGAGTAGTACCGCTAGCATTAC	87
myoc	CAAAAAGACAACAGTTCAGACC	GACCACAGCCGTCAACTC	149
lgsn	AGACAGTTCCAGCTCGATTCT	CTGCACTTGCATGGTCAAC	117
vangl2	CCCAGTCTACAATCCTGCTTTA	GACTGACCCGTCGAGTTATTAG	120

Supplementary Table S2. Top-50 downregulated genes in altered eyes of old (two years) male myoc transgenic zebrafish identified in the transcriptomic study. *DEGs* that coincided in the two comparisons of the ocular transgenic transcriptome (Tg) with each of the two wild type (WT) ocular transcriptomes were selected. Differential expression threshold: foldchange <-2; read count>20. p-value<0.05.

Gene symbol	Gene ID	Identification	Fold-change (Tg vs WT)	p-value	Read count	
					Tg/+	WT (mean)
crybgx	553474	crystallin beta gamma X	-217.3	1.5E-50	420	76915
bfsp2	494090	beaded filament structural protein 2, phakinin	-206.4	6.1E-56	835	145492
lenep	100002665	lens epithelial protein	-153.8	1.8E-49	486	63188
crygm5	474328	crystallin, gamma M5	-130.3	1.2E-31	365	40142
crygm2c	493628	crystallin, gamma M2c	-109.0	5.2E-22	1103	101352
crygm7	553967	crystallin, gamma M7	-104.3	3.7E-19	733	77831
clic5b	405833	chloride intracellular channel 5b	-95.0	1.1E-06	80	4589
lctlb	564250	lactase-like b	-71.8	5.9E-10	140	8510
lctla	437008	lactase-like a	-59.6	4.4E-10	979	49309
zgc:153704	767759	zgc:153704	-48.4	4.1E-06	1857	7597
ggctb	406278	gamma-glutamylcyclotransferase b	-45.2	5.0E-99	613	23417
lrrc20	492485	leucine rich repeat containing 20	-41.2	2.2E-82	579	20104
tgm1l2	793096	transglutaminase 1 like 2	-41.1	2.8E-06	76	2682
mipa	445140	major intrinsic protein of lens fiber a	-39.7	5.4E-90	1277	42803
si:ch73-335l21.1	794738	si:ch73-335l21.1, transcript variant X3	-38.9	8.1E-79	248	8141
zgc:112285	561476	zgc:112285	-31.6	1.6E-72	1482	39458
crybb1l3	566891	crystallin, beta B1, like 3	-30.2	7.7E-73	253	6454
pah	378962	phenylalanine hydroxylase	-29.5	4.6E-21	48	1192
LOC101884821	101884821	desmoplakin-like	-28.5	1.2E-43	40	971
ddx19a	563448	zgc:193690	-27.7	1.9E-42	36	845
lim2.2	572341	lens intrinsic membrane protein 2.2	-26.0	1.5E-63	832	18204
fabp11a	447944	fatty acid binding protein 11a	-25.4	5.9E-66	4642	99495
tspan33b	567512	tetraspanin 33b	-24.6	6.9E-63	380	7885
sprn2	100006310	shadow of prion protein 2	-24.0	2.7E-43	22	450
cryba1a	436683	crystallin, beta A1a	-23.6	1.4E-60	14878	29600
ppt2a.3	406830	zgc:55413	-23.1	1.5E-32	22	413
jac8	796228	jacalin 8	-22.5	8.5E-59	1968	74735
fosb	492346	FBJ murine osteosarcoma viral oncogene homolog B	-22.4	5.1E-65	173	3296
si:ch211-14c7.2	571950	si:ch211-14c7.2, transcript variant X11	-19.7	2.1E-63	656	10910

Gene symbol	Gene ID	Identification	Fold-change (Tg vs WT)	p-value	Read count	
					Tg/+	WT (mean)
lim2.1	573354	lens intrinsic membrane protein 2.1	-19.4	3.8E-61	556	9102
jdp2b	436766	Jun dimerization protein 2b	-19.2	4.1E-50	54	878
zgc:153031	557552	zgc:153031	-19.1	7.9E-49	193	3119
lim2.3	436860	lens intrinsic membrane protein 2.3	-17.9	5.8E-51	556	8426
mctp2b	562526	multiple C2 domains, transmembrane 2b, transcript variant X2	-17.3	3.6E-45	68	997
crybb1	114418	crystallin, beta B1	-17.1	6.4E-50	8077	116166
serpinb1	436926	serpin peptidase inhibitor, clade B (ovalbumin), member 1	-17.0	2.6E-50	5259	75518
crybb1l1	553473	crystallin, beta B1, like 1	-16.9	2.9E-53	423	6018
si:ch211-69b7.6	556640	si:ch211-69b7.6	-16.3	3.9E-46	837	11595
lim2.5	541395	lens intrinsic membrane protein 2.5	-16.2	6.1E-51	945	12901
dsc2l	560091	desmocollin 2 like	-16.0	1.6E-29	1236	16878
myocd	100331725	myocardin, transcript variant X4	-15.9	3.2E-39	43	579
fam234a	796439	family with sequence similarity 234, member A, transcript variant X1	-14.9	1.1E-52	440	5572
LOC101883471	101883471	specifically androgen-regulated gene protein-like	-14.8	1.2E-24	53	672
havcr2	100536120	hepatitis A virus cellular receptor 2, transcript variant X2	-14.6	7.2E-29	75	789
tuft1b	100005886	tuftelin 1b	-14.3	2.4E-26	34	416
fbxl16	797939	F-box and leucine-rich repeat protein 16	-13.4	2.3E-35	71	805
cmtm4	436765	CKLF-like MARVEL transmembrane domain containing 4	-13.3	1.6E-24	21	238
pcdh20	100007362	protocadherin 20	-13.2	1.7E-22	36	408
jcada	100001168	junctional cadherin 5 associated a, transcript variant X1	-13.1	1.4E-54	758	8504
capn12	799247	calpain 12	-12.4	6.9E-38	363	3834

Supplementary Table S32. Top-50 upregulated genes in altered eyes of old (two years) male *myoc* transgenic zebrafish identified in the transcriptomic study. DEGs that coincided in the two comparisons of the ocular transgenic transcriptome (Tg) with each of the two wild type (WT) ocular transcriptomes were selected. Differential expression threshold: foldchange <-2; read count>20. p-value<0.05.

Gene symbol	Gene ID	Identification	Fold-change (Tg vs. WT)	p-value	Read count	
					Tg/+	WT (mean)
Ccdc24	541495	coiled-coil domain containing 24	54.1	4.9E-08	2586	315
col7a1l	559516	collagen type VII alpha 1-like	28.6	4.7E-07	1562	72
angptl3	010817	angiopoietin-like 3	28.5	2.4E-11	1624	77
tnni2a.4	494164	troponin I type 2a (skeletal, fast), tandem duplicate 4	26.5	3.9E-05	3066	726
Pvalb2	58028	parvalbumin 2	24.6	5.9E-09	7218	1283
fj08f03	030131	si:ch211-153b23.4	23.9	2.4E-16	16278	6619
pvalb1	402805	parvalbumin 1	21.3	3.0E-10	7218	1238
ptk2ba	768185	protein tyrosine kinase 2 beta, a, transcript variant X2	17.6	6.1E-06	500	25
snx19a	550357	sorting nexin 19a	17.2	9.9E-05	4333	213
zgc:112332	678602	zgc:112332	16.6	7.8E-11	6499	337
tpma	445049	alpha-tropomyosin	16.5	1.7E-06	9638	1766
irg1l	100004274	immunoresponsive gene 1, like	15.3	1.1E-09	2381	133
rnh1	569436	ribonuclease/angiogenin inhibitor 1	13.6	3.3E-09	772	51
zgc:172122	571844	zgc:172122	13.1	2.1E-12	426	29
mylpfb	447930	myosin light chain, phosphorylatable, fast skeletal muscle b	12.9	1.7E-05	3485	725
cyp39a1	564475	cytochrome P450, family 39, subfamily A, polypeptide 1	12.7	3.9E-05	481	32
Cb252	565454	si:ch211-153b23.5	12.5	6.2E-21	1450	107
chchd3b	325751	coiled-coil-helix-coiled-coil-helix domain containing 3b	11.5	8.2E-04	563	47
myhz1.2	799300	myosin, heavy polypeptide 1.2, skeletal muscle	10.8	5.9E-07	8501	1772
crygm2d17	561929	crystallin, gamma M2d17	10.6	2.3E-04	699	180
crygm2d20	100000175	crystallin, gamma M2d20	10.2	2.4E-05	489	116
crygm2d16	798701	crystallin, gamma M2d16	10.1	4.9E-15	574	143
gtf3c3	553318	general transcription factor IIIC, polypeptide 3	9.7	3.0E-17	4978	436
si:ch211-202m22.1	100536303	si:ch211-202m22.1	8.9	1.4E-05	363	35
spice1	494093	spindle and centriole associated protein 1	8.6	2.8E-03	526	55
nos2b	563654	nitric oxide synthase 2b, inducible	8.5	4.3E-06	689	78

Gene symbol	Gene ID	Identification	Fold-change (Tg vs. WT)	p-value	Read count	
					Tg/+	WT (mean)
col28a1a	555428	collagen, type XXVIII, alpha 1a	8.2	1.2E-16	2128	226
tnnc2	58082	troponin C type 2 (fast)	8.1	5.7E-25	2569	565
smyhc1	321552	slow myosin heavy chain 1	7.9	3.4E-16	894	123
tmem56b	449652	transmembrane protein 56b	7.9	4.4E-11	3951	432
myl10	550569	myosin, light chain 10, regulatory	7.8	2.8E-13	864	178
myhz1.3	100008070	ns:zf-e68	7.6	1.4E-08	8501	1090
synpo	100536545	synaptopodin, transcript variant X2	7.3	5.1E-50	386	58
si:ch211-103a14.5	100330921	si:ch211-103a14.5	7.2	7.6E-06	626	97
dcdc2b	563259	doublecortin domain containing 2B	7.0	7.2E-10	1376	166
qpct	795685	glutaminyl-peptide cyclotransferase	6.9	2.9E-04	270	35
myl1	336165	myosin, light chain 1, alkali; skeletal, fast	6.8	4.7E-08	1630	378
card9	796425	caspase recruitment domain family, member 9	6.8	1.5E-16	394	49
ccdc180	100538012	coiled-coil domain containing 180, transcript variant X3	6.6	9.9E-06	242	36
gnb1l	541381	guanine nucleotide binding protein (G protein), beta polypeptide 1-like	6.5	3.5E-07	251	33
spag9a	558833	sperm associated antigen 9a	6.5	5.2E-11	387	54
si:ch211-163l21.8	449761	si:ch211-163l21.8	6.4	6.1E-06	315	55
actc1b	58114	actin, alpha, cardiac muscle 1b	6.4	6.3E-08	25146	5664
si:ch211-256m1.8	795494	si:ch211-256m1.8	6.3	1.6E-07	832	112
cfap70	564542	si:dkey-254e1.1	6.3	8.3E-08	720	103
ccdc15	100333818	coiled-coil domain containing 15	6.2	2.3E-05	319	56
crygm2d3	790908	crystallin, gamma M2d3	6.1	8.2E-05	589	154
ptprbl	100333658	protein tyrosine phosphatase, receptor type, B, like	6.1	4.8E-09	427	91
cyp11c1	791124	cytochrome P450, family 11, subfamily C, polypeptide 1	6.1	4.5E-05	235	34

Supplementary Table S4. Confirmation by qPCR of selected DEGs identified in the transcriptome analysis.

Gene	Gene ID (GeneBank)	Transcriptome		qPCR	
		Fold-change	p-value	Fold-change ($2^{-\Delta\Delta CT}$)	p-value
Angptl3	010817	28.5	2,4E-11	15.0	0.002
Arhgef40	100151026	-3.4	2,2E-08	-1.8	0.009
Bfsp2	494090	-206.4	6,1E-56	-9.1	0.001
Cav2	415240	-3.5	1,9E-12	-23.4	0.002
Ccdc24	541495	54.1	4,9E-08	56.8	0.005
crybgx	553474	-217.3	1,5E-50	-76.9	0.004
lgsn	567024	-156.5	3,9E-16	4.4	0.001
Vangl2	245949	-4.8	3,3E-08	-1.6	0.001

Supplementary Table S5. Gene ontology (GO) enrichment analysis of the top-50 up and downregulated genes in the transcriptomic study. p-value <0.05. The analysis was carried out using the Epistemic artificial inteligence web-based software platform. B: BioPlanet 2019; EPC: Elsevier Pathway Collection; FDR: false discovery rate. KEGG; Kyoto encyclopedia of genes and genomes; Overlap count: number of uploaded genes that belong to the different functional groups. Gene set count: total number of genes in the functional group.

Functional group	Database	Overlap count	Gene set count	FDR adjust. p-value	Mapped gene symbols	Uploaded genes
Proteins Involved in Cataract	EPC	12	94	3,9E+04	*BFSP2, CRYBA1, CRYBB1, CRYBB2, CRYBB3, CRYGB, CRYGC, CRYGD, FOSB, IRS1, LIM2, MIP*	*bfsp2, cryba1a, crybb1, crybb1l1, crybb1l3, crygm2c, crygm2d16, crygm2d17, crygm2d20, crygm2d3, crygm5, crygm7, fosb, lim2.1, lim2.3, lim2.5, mipa, si:ch73-335l21.1*
Cataract Congenital	EPC	8	19	3,9E+04	*BFSP2, CRYBB1, CRYBB2, CRYGB, CRYGC, CRYGD, LIM2, MIP*	*bfsp2, crybb1, crybb1l1, crybb1l3, crygm2c, crygm2d16, crygm2d17, crygm2d20, crygm2d3, crygm5, crygm7, lim2.1, lim2.3, lim2.5, mipa*
Genes With Mutations Associated with Cataract	EPC	8	21	3,9E+04	*BFSP2, CRYBB1, CRYBB2, CRYBB3, CRYGC, CRYGD, LIM2, MIP*	*bfsp2, crybb1, crybb1l1, crybb1l3, crygm2c, crygm2d16, crygm2d17, crygm2d20, crygm2d3, crygm5, crygm7, lim2.1, lim2.3, lim2.5, mipa*
Striated Muscle Contraction	B	6	38	2,8E+08	*ACTC1, MYL1, TNNC2, TNNI2, TPM1, TPM3*	*actc1b, myl1, tnnc2, tnni2a.4, tpma*
Muscle Contraction	B	5	49	2,6E+11	*MYL1, TNNC2, TNNI2, TPM1, TPM3*	*myl1, tnnc2, tnni2a.4, tpma*
Steroids Induced Cataract	EPC	5	59	2,4E-04	*BFSP2, CRYBB2, CRYGD, IRS1, MIP*	*bfsp2, crybb1l3, crygm2c, crygm2d16, crygm2d17, crygm2d20, crygm2d3, crygm5, crygm7, mipa, si:ch73-335l21.1*
Hypertrophic Cardiomyopathy	EPC	5	88	1,4E-03	*ACTC1, IRS1, MYH7, PTK2B, TPM1*	*actc1b, ptk2ba, si:ch73-335l21.1, smyhc1, tpma*
Integrins in Angiogenesis	B	4	74	2,6E-03	*ANGPTL3, COL7A1, IRS1, PTK2B*	*angptl3, col7a1l, ptk2ba, si:ch73-335l21.1*
Cardiac Muscle Contraction	B	4	83	3,0E-03	*ACTC1, MYH7, TPM1, TPM3*	*actc1b, smyhc1, tpma*
Dilated Cardiomyopathy	B	4	100	4,9E-03	*ACTC1, MYH7, TPM1, TPM3*	*actc1b, smyhc1, tpma*
Tight Junction	B	4	133	1,0E-02	*MYH4, MYH7, MYL10, MYLPF*	*myhz1.2, myhz1.3, myl10, mylpfb, smyhc1*

Functional group	Database	Overlap count	Gene set count	FDR adjust. p-value	Mapped gene symbols	Uploaded genes
Diabetes Induced Cataract	EPC	4	18	4,5E+11	*CRYGB, CRYGC, CRYGD, MIP*	*crygm2c, crygm2d16, crygm2d17, crygm2d20, crygm2d3, crygm5, crygm7, mipa*
Age-Related Cataract	EPC	4	23	1,0E-04	*CRYGB, CRYGC, CRYGD, MIP*	*crygm2c, crygm2d16, crygm2d17, crygm2d20, crygm2d3, crygm5, crygm7, mipa*
Proteins Involved in Hypertrophic Cardiomyopathy	EPC	4	65	2,8E-03	*ACTC1, FOSB, MYH7, TPM1*	*actc1b, fosb, smyhc1, tpma*
Dilated Cardiomyopathy	EPC	4	69	3,1E-03	*ACTC1, IRS1, MYH7, TPM1*	*actc1b, si:ch73-335l21.1, smyhc1, tpma*
Proteins Involved in Dilated Cardiomyopathy	EPC	4	166	3,6E-02	*ACTC1, MYH7, MYOCD, TPM1*	*actc1b, myocd, smyhc1, tpma*
Cardiac Muscle Contraction	KEGG	4	78	1,4E-03	*ACTC1, MYH7, TPM1, TPM3*	*actc1b, smyhc1, tpma*
Hypertrophic Cardiomyopathy (HCM)	KEGG	4	83	1,4E-03	*ACTC1, MYH7, TPM1, TPM3*	*actc1b, smyhc1, tpma*
Dilated Cardiomyopathy	KEGG	4	89	1,4E-03	*ACTC1, MYH7, TPM1, TPM3*	*actc1b, smyhc1, tpma*
Adrenergic Signaling in Cardiomyocytes	KEGG	4	144	5,1E-03	*ACTC1, MYH7, TPM1, TPM3*	*actc1b, smyhc1, tpma*
Leukocyte Transendothelial Migration	B	3	117	3,5E-02	*MYL10, MYLPF, PTK2B*	*myl10, mylpfb, ptk2ba*
Oxygen Shortage in Myocardial Ischemia	EPC	3	20	1,4E-03	*ACTC1, MYH7, TPM1*	*actc1b, smyhc1, tpma*
Sarcomere in Cardiomyocyte in Dilated Cardiomyopathy	EPC	3	25	2,4E-03	*ACTC1, MYH7, TPM1*	*actc1b, smyhc1, tpma*
Ca2+ Toxicity in Lens Cells	EPC	3	27	2,7E-03	*CRYGB, CRYGC, CRYGD*	*crygm2c crygm2d16, crygm2d17, crygm2d20, crygm2d3, crygm5, crygm7*

Functional group	Database	Overlap count	Gene set count	FDR adjust. p-value	Mapped gene symbols	Uploaded genes
Sarcomere Disorganization and Intracellular Calcium Overload	EPC	3	29	2,8E-03	*ACTC1, MYH7, TPM1*	*actc1b, smyhc1, tpma*
TRPM4/6/7/8 Signaling in Arterial Hypertension (Hypothesis)	EPC	3	39	5,8E-03	*MYOCD, PTK2B, TPM1*	*myocd, ptk2ba, tpma*
Cardiomyocyte Dysfunction	EPC	3	48	9,8E-03	*ACTC1, MYH7, TPM1*	*actc1b, smyhc1, tpma*
Cardiomyocyte Hypertrophy	EPC	3	65	1,8E-02	*IRS1, MYH7, PTK2B*	*ptk2ba, si:ch73-335l21.1, smyhc1*
Leukocyte Transendothelial Migration	KEGG	3	114	1,5E-02	*MYL10, MYLPF, PTK2B*	*myl10, mylpfb, ptk2ba*
Cytochrome P450 Metabolism of Endogenous Sterols	B	2	15	9,6E-03	*CYP11B1, CYP39A1*	*cyp11c1, cyp39a1*
Smooth Muscle Contraction	B	2	22	1,4E-02	*TPM1, TPM3*	*tpma*
Visual Signal Transduction: Cones	B	2	23	1,4E-02	*LOC118142757, RDH12*	*si:ch211-103a14.5, zgc:112332*
Visual Signal Transduction: Rods	B	2	23	1,4E-02	*LOC118142757, RDH12*	*si:ch211-103a14.5, zgc:112332*
Steroid HormoneVitamin A and Vitamin D Metabolism	B	2	36	3,0E-02	*CYP11B1, RDH11*	*cyp11c1, zgc:112332*

Functional group	Database	Overlap count	Gene set count	FDR adjust. p-value	Mapped gene symbols	Uploaded genes
Rod Visual Cycle Impairment in Retinitis Pigmentosa	EPC	2	16	1,1E-02	*RDH11, RDH12*	*zgc:112332, zgc:112332*
Visual Cycle in Retinal Rods	EPC	2	16	1,1E-02	*RDH11, RDH12*	*zgc:112332, zgc:112332*
NTRK1/2/3 -> Acetylcholine Production	EPC	2	17	1,2E-02	*IRS1, PTK2B*	*ptk2ba, si:ch73-335l21.1*
Microglia and Motor Neuron Interaction Dysregulation	EPC	2	26	2,5E-02	*CD200, PTK2B*	*ptk2ba, zgc:172122*
Aldosterone Synthesis Chronic Regulation	EPC	2	31	3,1E-02	*CYP11B1, PTK2B*	*cyp11c1, ptk2ba*
Estrogens/Esr1 Non-Genomic Signaling	EPC	2	32	3,1E-02	*IRS1, PTK2B*	*ptk2ba, si:ch73-335l21.1*
IGF1R -> MEF/MYOD/MYOG Signaling	EPC	2	32	3,1E-02	*IRS1, PTK2B*	*ptk2ba, si:ch73-335l21.1*
Vitamin a (Retinol) Metabolism and Visual Cycle	EPC	2	32	3,1E-02	*RDH11, RDH12*	*zgc:112332, zgc:112332*
Folate Biosynthesis	KEGG	2	15	5,1E-03	*DHFR, DHFR2*	*zgc:153031, zgc:153031*
One Carbon Pool by Folate	KEGG	2	20	6,1E-03	*DHFR, DHFR2*	*zgc:153031, zgc:153031*
Antifolate Resistance	KEGG	2	31	1,2E-02	*DHFR, DHFR2*	*zgc:153031, zgc:153031*
Retinol Metabolism	KEGG	2	65	4,0E-02	*RDH11, RDH12*	*zgc:112332, zgc:112332*

Supplementary Table S6. Gene ontology (GO) enrichment analysis of the top-50 up and downregulated genes in the transcriptomic study. p-value <0.05. The analysis was carried out with the web tool ShinyGO (N=differentially expressed genes/number of genes in the GO category).

N	High level GO category	Genes
12/98	Lens development in camera-type eye	*crybb1l1, crybgx, crygm7, cryba1a, crygm5, crybb1, crygm2d17, crygm2d16, crygm2d3, crygm2d20, crygm2c, mipa*
12/187	Visual perception	*crybb1l1, crybgx, crygm7, cryba1a, crygm5, crybb1, crygm2d17, crygm2d16, crygm2d3, crygm2d20, crygm2c, si:ch211-103a14.5*
12/195	Sensory perception of light stimulus	*crybb1l1, crybgx, crygm7, cryba1a, crygm5, crybb1, crygm2d17, crygm2d16, crygm2d3, crygm2d20, crygm2c, si:ch211-103a14.5*
13/361	Eye development	*crybb1l1, crybgx, crygm7, cryba1a, crygm5, crybb1, crygm2d17, crygm2d16, crygm2d3, crygm2d20 crygm2c, mipa, fabp11a*
13/361	Visual system development	*crybb1l1, crybgx, crygm7, cryba1a, crygm5, crybb1 crygm2d17, crygm2d16, crygm2d3, crygm2d20 crygm2c, mipa, fabp11a*
12/309	Camera-type eye development	*crybb1l1, crybgx, crygm7, cryba1a, crygm5, crybb1 crygm2d17, crygm2d16, crygm2d3, crygm2d20 crygm2c, mipa*
13/411	Sensory perception	*crybb1l1, crybgx, crygm7, cryba1a, crygm5, crybb1 crygm2d17, crygm2d16, crygm2d3, crygm2d20 crygm2c, clic5b, si:ch211-103a14.5*
13/437	Sensory system development	*crybb1l1, crybgx, crygm7, cryba1a, crygm5, crybb1 crygm2d17, crygm2d16, crygm2d3, crygm2d20 crygm2c, mipa, fabp11a*
13/484	Sensory organ development	*crybb1l1, crybgx, crygm7, cryba1a, crygm5, crybb1 crygm2d17, crygm2d16, crygm2d3, crygm2d20 crygm2c, mipa, fabp11a*
16/885	System process	*crybb1l1, tnni2a.4, crybgx, crygm7, cryba1a, crygm5, crybb1, crygm2d17, tnnc2, crygm2d16 crygm2d3, crygm2d20, crygm2c, clic5b si:ch211-103a14.5, smyhc1*
13/559	Nervous system process	*crybb1l1, crybgx, crygm7, cryba1a, crygm5, crybb1 crygm2d17, crygm2d16, crygm2d3, crygm2d20 crygm2c, clic5b, si:ch211-103a14.5*
3/26	Skeletal muscle contraction	*tnni2a.4, tnnc2, smyhc1*
3/27	Multicellular organismal movement	*tnni2a.4, tnnc2, smyhc1*
3/27	Musculoskeletal movement	*tnni2a.4, tnnc2, smyhc1*
15/1872	Animal organ development	*crybb1l1, crybgx, crygm7, cryba1a, crygm5, crybb1 crygm2d17, crygm2d16, crygm2d3, crygm2d20 crygm2c, ptk2ba, mipa, capn12, fabp11a*
3/54	Striated muscle contraction	*tnni2a.4, tnnc2, smyhc1*
22/4051	Multicellular organismal process	*crybb1l1, ptk2ba, tnni2a.4, crybgx, crygm7, cryba1a crygm5, crybb1, crygm2d17, tnnc2, crygm2d16 crygm2d3, jcada, crygm2d20, crygm2c, clic5b, lenep si:ch211-103a14.5, mipa, smyhc1, capn12, fabp11a*

1.2 Supplementary Figures

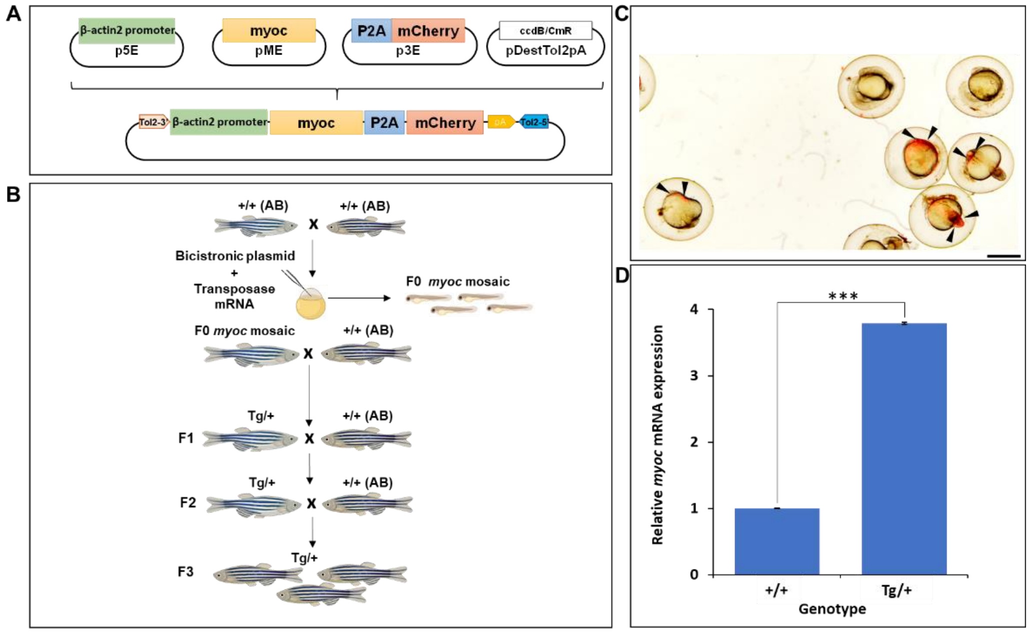

Supplementary Figure S1. Generation of a *myoc* transgenic line [*Tg(actb1:myoc-2A-mCherry)*] using the gateway system and transposon-mediated transgenesis**.** (A) Combination of gateway vectors employed to create the bicistronic transgene cassette, which co-express myocilin and the reporter protein mCherry. pA: poly-A signal; P2A: porcine teschovirus-1 autolytic peptide 2A; p5E: plasmid 5'-entry; pE: plasmid middle-entry; p3E: plasmid 3'-entry; Tol2: recombination Tol2 sites. (B) Stepwise procedure followed to establish the transgenic zebrafish line. The cartoon was created with Biorender. (C) Detection of mCherry fluorescence in F0 microinjected embryos (24 hpf). Red fluorescence and brightfield images are merged. Arrowheads: mCherry fluorescence. (D) Increased *myoc* mRNA level in the transgenic zebrafish line. RNA was purified using pools of 50 fluorescent zebrafish larvae (144 hpf) obtained by crossing F3 transgenic and wild type zebrafish. *Myoc* mRNA level were measured by qRT-PCR. Values represent the average of two independent biological experiments performed in triplicate. ***: p<0.001, Student's t-test. +/+: wild type; Tg/+: transgenic.

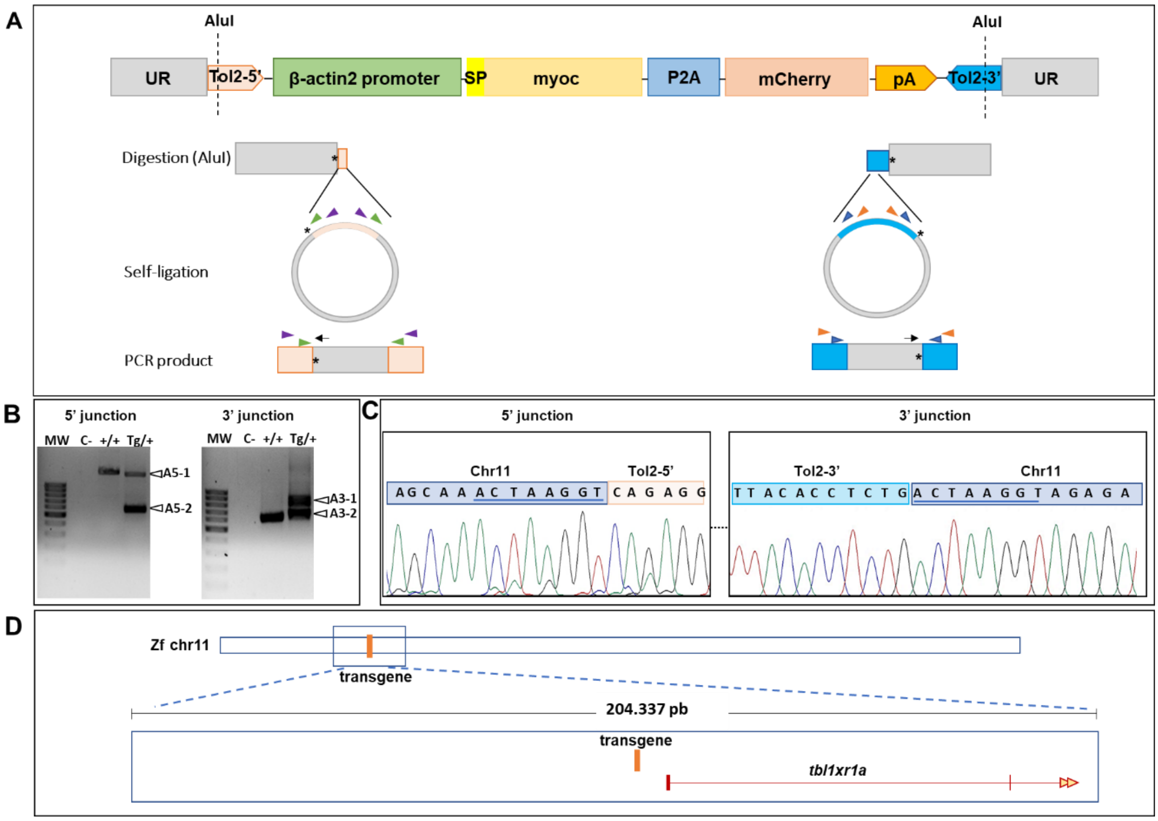

Supplementary Figure S2. Identification of the transgenic integration site. (A) Diagram of the nested PCR process. *Alu*I restriction target. Tol2 specific primers (arrowheads). Black arrows: sequencing primers from chromosome 11. Purple and orange arrowheads: primers used for the first PCR; green and blue arrowheads: primers used for the second PCR. Asterisk: insertion site. UR: unknown region. SP: signal peptide. P2A: porcine teschovirus-1 autolytic peptide 2A. (B) Agarose gel electrophoresis of the nested PCR amplicons. (C) Representative Sanger sequencing of the amplicons obtained by nested PCR. MW: molecular weight marker; +/+: wild-type; Tg/+: transgenic; C-: negative control. (D) Chromosomal localization of the insertion site of the bicistronic transgene. Zf Chr11: zebrafish chromosome 11.

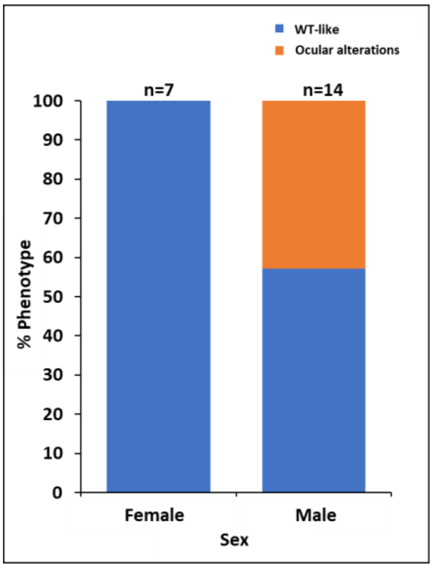

Supplementary Figure S3. Ocular phenotype analysis in old (two years old) *myocilin* transgenic zebrafish. Ocular alterations were not observed in females.

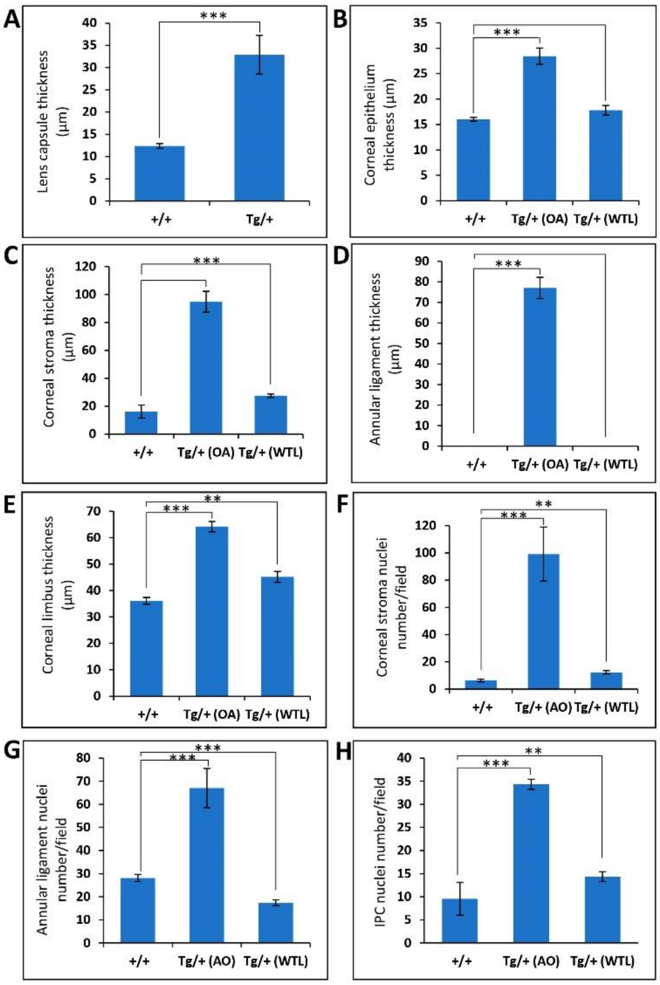

Supplementary Figure S4. Quantification of different ocular parameters in old (two years old) myocilin transgenic zebrafish. Thickness of the lens capsule(A), corneal epithelium (B), corneal stroma (C), annular ligament in the central cornea (D), and corneal limbus (E). Nuclei number in the corneal stroma (F), annular ligament (G) and iris pigmented cells (H). Thickness and number of nuclei was counted in four microscopic fields per eye. Hematoxilin-eosin-stained tissue sections were used for thickness measurements. Nuclei were counted in DAPI-stained tissue sections. Tissue sections from four eyes of each phenotype were used for the analyses. ***: $p<0.001$; **: $p<0.05$, Student's t-test. +/+: wildtype; OA: ocular alterations; Tg/+: transgenic; WTL: wild type-like.

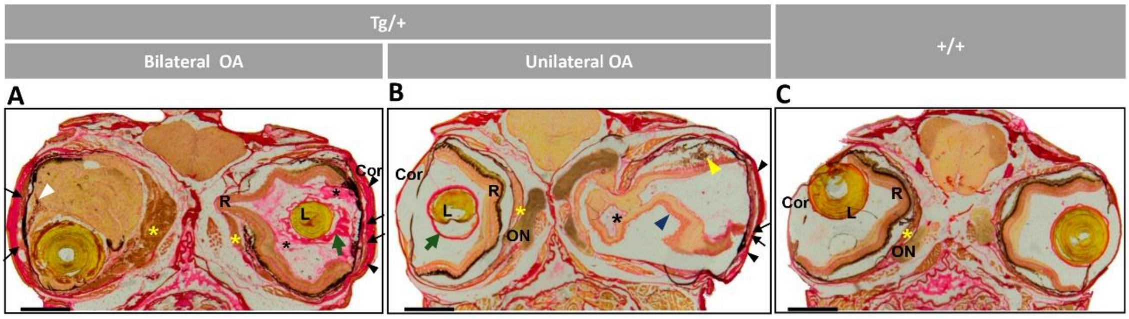

Supplementary Figure S5. Histology of head sections of adult male transgenic zebrafish (two years old). Sirius red tissue sections correspond to transgenic zebrafish with bilateral or unilateral macroscopic ocular alterations (A and B, respectively). Control tissue sections from same-age wild type zebrafish (C). Tissue sections are consecutive with those shown in **Figure 2**. Scale bar represents 800 µm. Black arrows: Tight contact between the cornea and iris; black arrowheads: thickening of the corneal stroma; green arrow: thickening of lens capsule; blue arrowhead: overgrowth and folding of the retina; yellow arrowhead: degeneration of photoreceptors; black asterisks: accumulation of vitreous material; yellow asterisks: overgrowth of the choroid body; Cor: cornea; OA: ocular alterations; ON: optic nerve; L: lens; R: retina; +/+: wild type; Tg/+: transgenic.

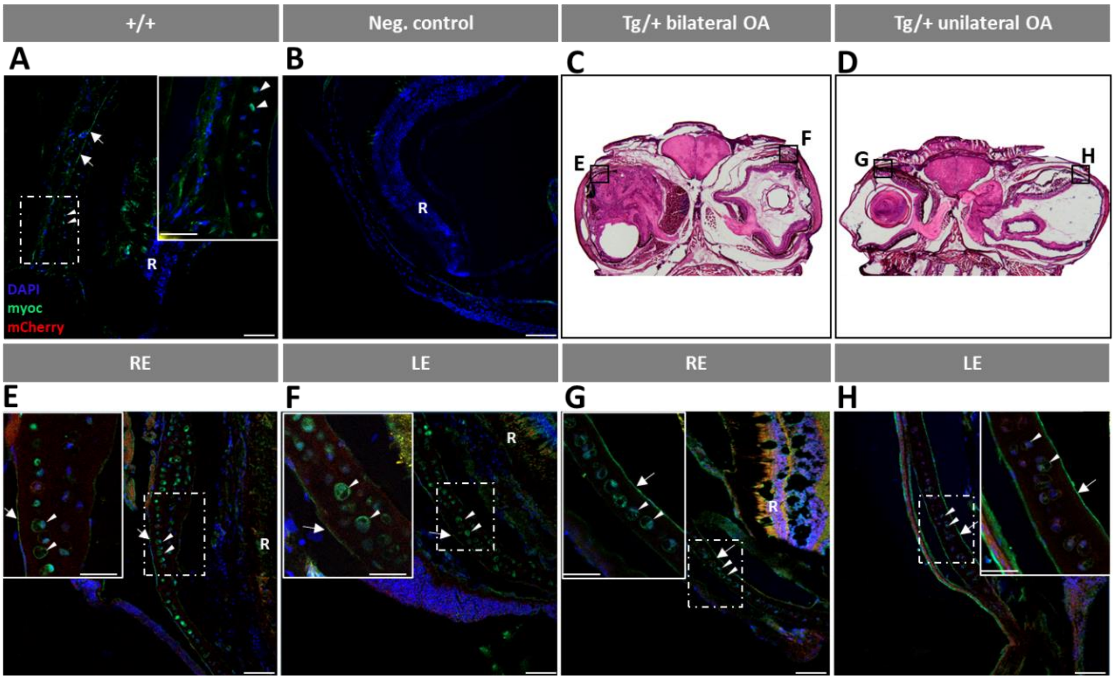

Supplementary Figure S6. Expression of myocilin and mCherry in the scleral cartilage of old (two years) myoc transgenic zebrafish. Tissue sections were incubated with a chicken anti-myocilin (TNT) (A, E-H) (green signal) or with only the secondary antibody as a negative control (B). Tissue sections shown in panels (C and D) indicate the regions analysed by immunohistochemistry. Scale bars: 50 μm. White arrows: Perichondrium immunoreactivity; white arrowheads: chondrocytes immunoreactivity; R: retina. Tg/+: transgenic; +/+: wild type. The images are representative of the results observed in two fishes.

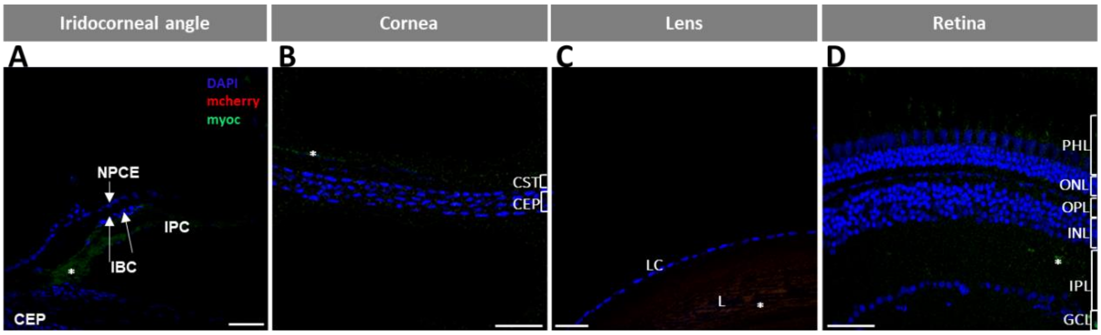

Supplementary Figure S7. Negative controls of the immunohistochemical myoc analysis in the old (two years) *myoc* transgenic zebrafish shown in **Figures 5 and 6**. Controls consisted of wild-type tissue sections incubated with a chicken preimmune anti-myocilin antibody (TNT), followed by the secondary antibody. Scale bars: 50 µm. Asterisks: autofluorescence. NPCE: Non-pigmented ciliary epithelium; IBC: Iris blood cells; IPC: iris pigment cells; CEP: Corneal epithelium; CST: corneal stroma; L: lens; LC: lens capsule; GCL: ganglion cell layer; IPL: inner plexiform layer; INL; inner nuclear layer; OPL: outer plexiform layer; ONL; outer nuclear layer; PHL: photoreceptor layer.

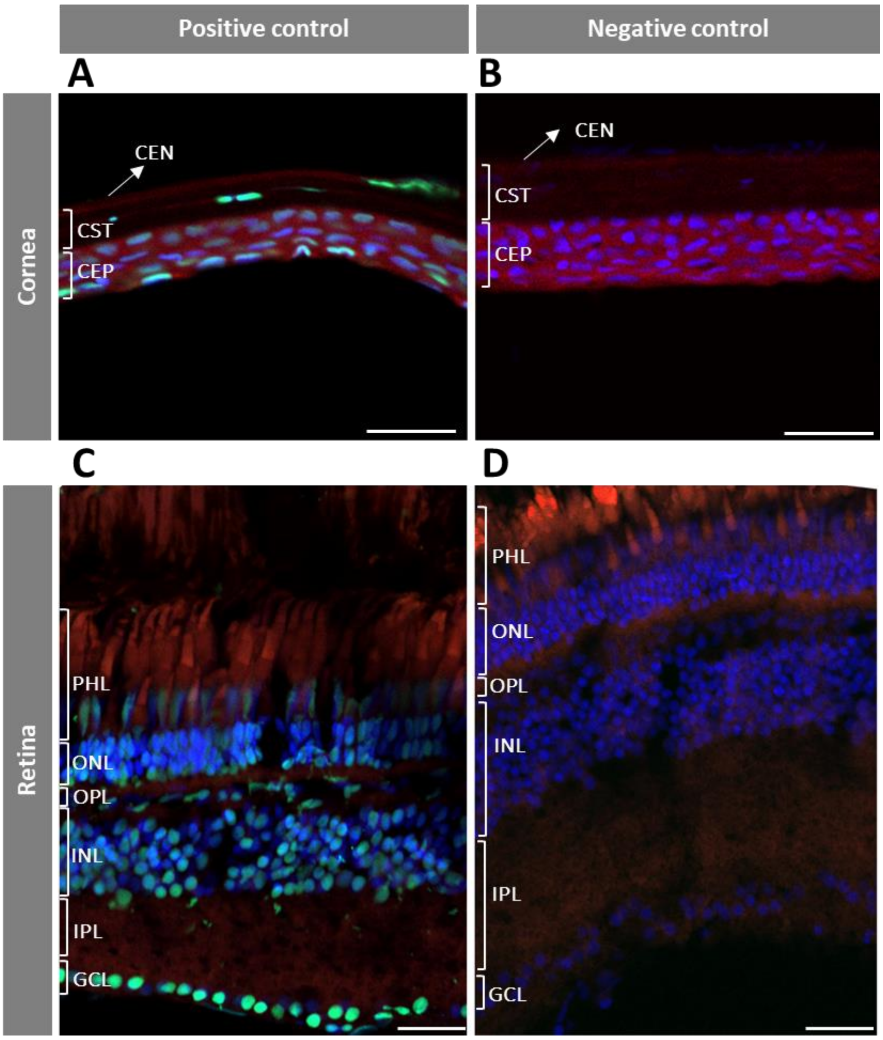

Supplementary Figure S8. Positive and negative controls of the TUNEL assay in the 2-year adult zebrafish shown in **Figure 7**. Retinal sections of adult wild-type zebrafish incubated with DNase I (A and C) or with only the labelling solution (B and D) were used as a positive and negative controls, respectively. Note the presence of positive cells in different cornea and retina layers. Scale bars: 50 μm in panels A and B and 25 μm in panels C and D. CEP: corneal epithelium; CST: corneal stroma; CEN: corneal endothelium; GCL: ganglion cell layer; IPL: inner plexiform layer; INL: inner nuclear layer; OPL: outer plexiform layer; ONL; outer nuclear layer; PHL: photoreceptor layer.

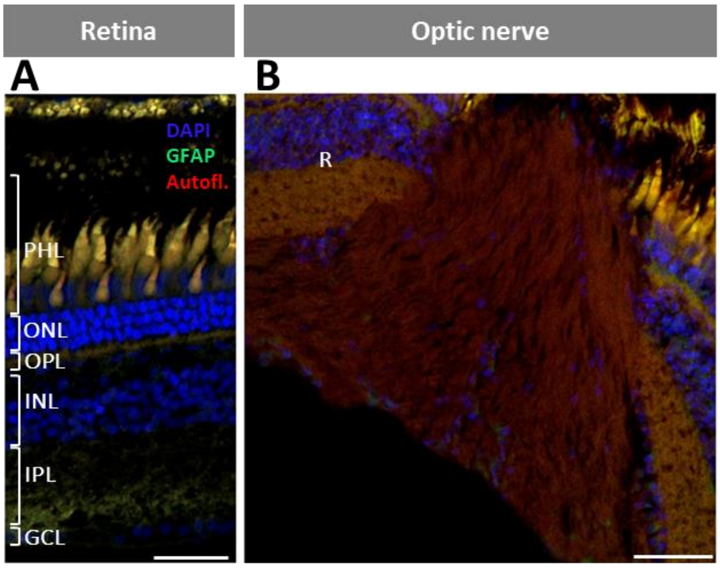

Supplementary Figure S9. Negative controls of the Immunohistochemical GFAP analysis in the 2-year adult zebrafish shown in **Figure 8**. Controls consisted of tissue sections of wild type incubated only with the secondary antibody. Scale bars: 25 µm. Autofl.: tissue autofluorescence was detected for image contrast and anatomical reference. GCL: ganglion cell layer; IPL: inner plexiform layer; INL: inner nuclear layer; OPL: outer plexiform layer; ONL; outer nuclear layer; PHL: photoreceptor layer; R: retina.

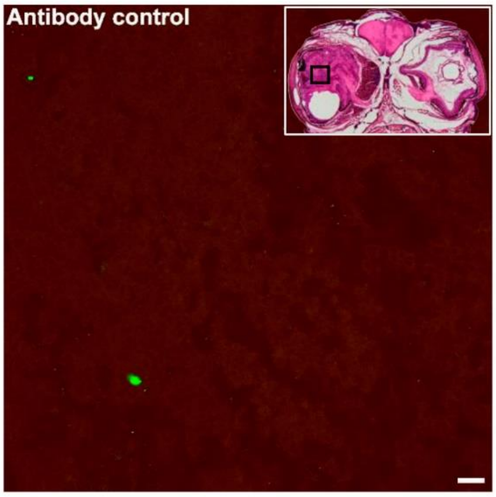

Supplementary Figure S10. Negative control of the immunohistochemical calretinin and Brn3a immunostaining in the 2-year adult zebrafish shown in **Figure 9**. The rectangle in the inset shows the approximate location of the region analyzed for the secondary antibody control in a representative tissue section. Controls consisted of ocular tissue sections only incubated with the secondary antibody mix (anti-rabbit Alexa-555 and anti-goat Alexa-633, Molecular Probes). Scale bar: 20 µm.

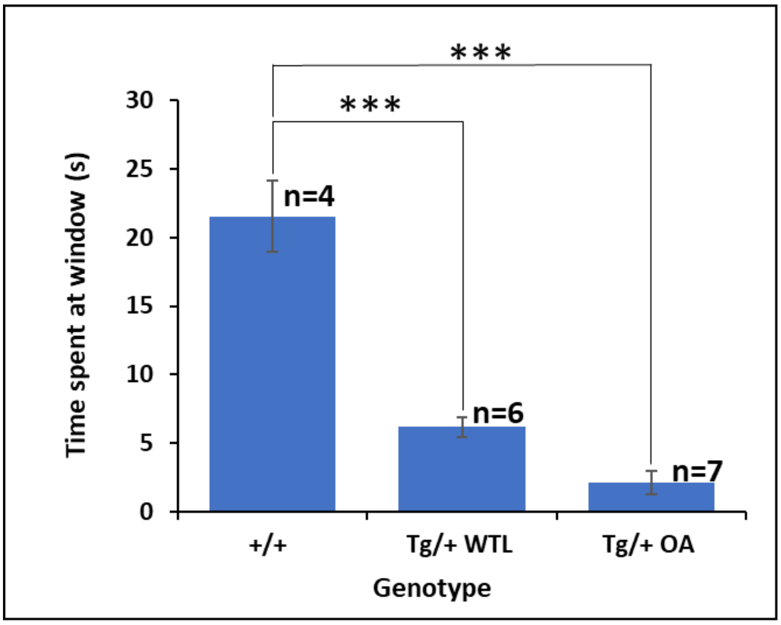

Supplementary Figure S11. Evaluation of visual function of old (two years) male transgenic zebrafish by a social preference assay. The time spent by the tested zebrafish at the window proximal to the social stimulus was measured in three intervals of one min and the mean value was calculated. Tg/+ WT-L: transgenic zebrafish with wild type like ocular phenotype. Tg/+ OA: transgenic zebrafish either with unilateral or bilateral ocular alterations; +/+: wild type. ***: $p<0.001$, Student's t-test.

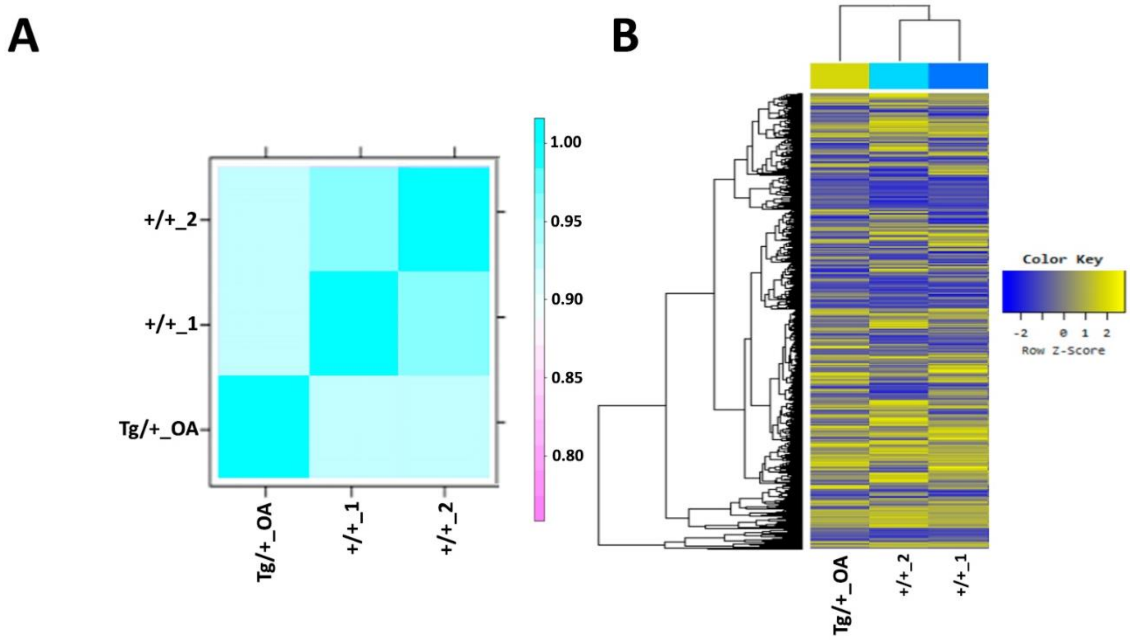

Supplementary Figure S12. (A) Correlation matrix showing the similarity between RNAseq replicas (Tg/+ OA eyes vs. +/+ eyes). The similarity between samples is obtained through Pearson's coefficient of sample's normalized value ($-1 \leq r \leq 1$). The closer the value is to 1, the more similar the samples are. (B) Heatmap of hierarchical clustering analysis of significant DEGs and replicas (Tg/+ OA eyes vs. +/+ eyes). Clustering analysis was done using Euclidean distance and complete linkage as a measure of similarity. The results show 3000 DEGs which satisfied a fold change > 2 or < -2 and a raw p value < 0.05. OA: ocular alterations; Tg/+: transgenic; +/+: wild-type.

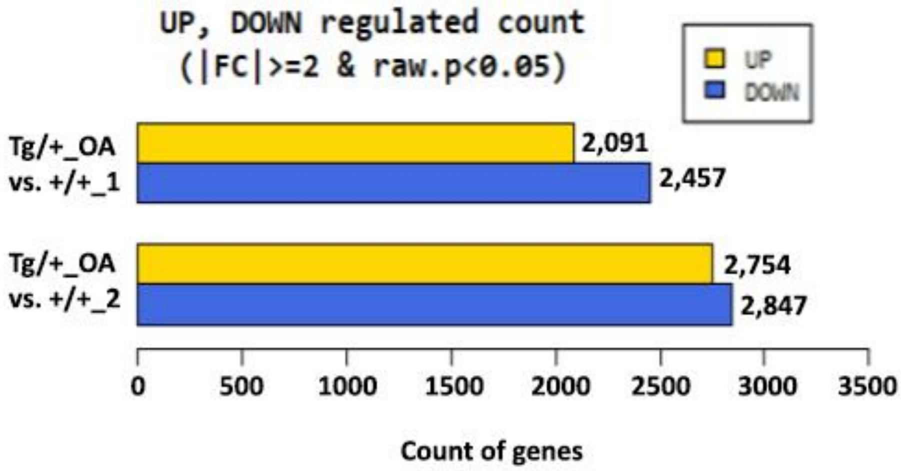

Supplementary Figure S13. Number of significant up and down regulated genes based on fold change of comparison pairs (*Tg/+ OA vs. +/+*). The results show DEGs which satisfied a fold change > 2 or < -2 and a raw p value < 0.05. OA: ocular alterations; Tg/+: transgenic; +/+: wild-type.

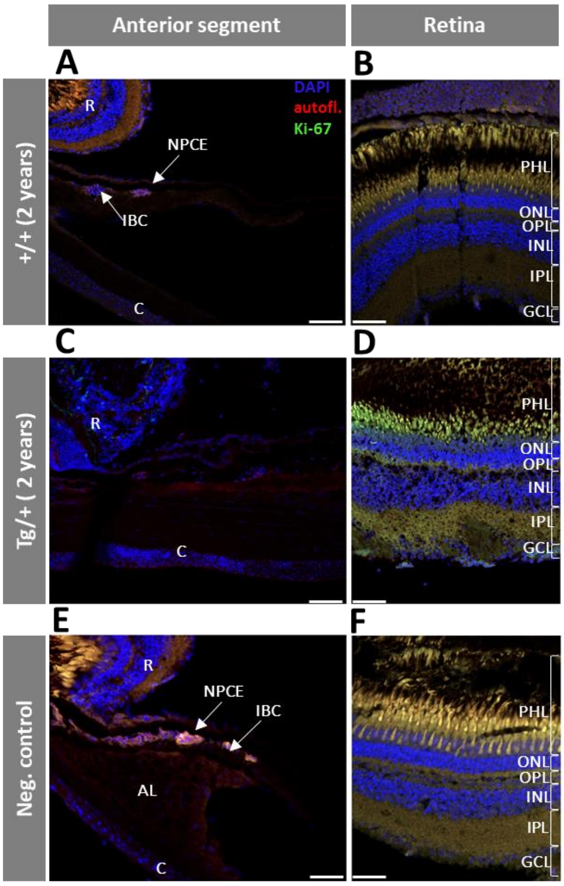

Supplementary Figure S14. Immunohistochemistry of the cell proliferation marker Ki-67 in the anterior segment and retina of old (two years) male transgenic and wild type (control) zebrafish. Histological sections were incubated with either an anti-Ki-67 primary antibody (A-D) and Cy2 donkey anti-rabbit secondary antibody or with only the secondary antibody as a negative control (E-F). Scale bars represent 50 μm. Autofl.: tissue autofluorescence was detected for image contrast and anatomical reference. AL: annular ligament; C: cornea; GCL: ganglion cell layer; IBC: iris blood cells; IPL: inner plexiform layer; INL: inner nuclear layer; NPCE: non pigment ciliary epithelium; OPL: outer plexiform layer; ONL: outer nuclear layer; PHL: photoreceptor layer. R: retina; Tg/+: transgenic; +/+: wild type. The images are representative of the results observed in two fishes of each genotype.
